# Practical algebraic calculus and Nullstellensatz with the checkers Pacheck and Pastèque and Nuss-Checker

**DOI:** 10.1007/s10703-022-00391-x

**Published:** 2022-04-11

**Authors:** Daniela Kaufmann, Mathias Fleury, Armin Biere, Manuel Kauers

**Affiliations:** 1 Institute for Formal Models and Verification, Johannes Kepler University, Linz, Austria; 2 Chair of Computer Architecture, Albert-Ludwigs-University, Freiburg, Germany; 3 Institute for Algebra, Johannes Kepler University, Linz, Austria

**Keywords:** Algebraic proof systems, Nullstellensatz proofs, Polynomial calculus, Gröbner basis, Arithmetic circuit verification, Isabelle/HOL

## Abstract

Automated reasoning techniques based on computer algebra have seen renewed interest in recent years and are for example heavily used in formal verification of arithmetic circuits. However, the verification process might contain errors. Generating and checking proof certificates is important to increase the trust in automated reasoning tools. For algebraic reasoning, two proof systems, Nullstellensatz and polynomial calculus, are available and are well-known in proof complexity. A Nullstellensatz proof captures whether a polynomial can be represented as a linear combination of a given set of polynomials by providing the co-factors of the linear combination. Proofs in polynomial calculus dynamically capture that a polynomial can be derived from a given set of polynomials using algebraic ideal theory. In this article we present the practical algebraic calculus as an instantiation of the polynomial calculus that can be checked efficiently. We further modify the practical algebraic calculus and gain LPAC (practical algebraic calculus + linear combinations) that includes linear combinations. In this way we are not only able to represent both Nullstellensatz and polynomial calculus proofs, but we are also able to blend both proof formats. Furthermore, we introduce extension rules to simulate essential rewriting techniques required in practice. For efficiency we also make use of indices for existing polynomials and include deletion rules too. We demonstrate the different proof formats on the use case of arithmetic circuit verification and discuss how these proofs can be produced as a by-product in formal verification. We present the proof checkers Pacheck, Pastèque, and Nuss-Checker. Pacheck checks proofs in practical algebraic calculus more efficiently than Pastèque, but the latter is formally verified using the proof assistant Isabelle/HOL. The tool Nuss-Checker is used to check proofs in the Nullstellensatz format.

**Supplementary Information:**

The online version contains supplementary material available at 10.1007/s10703-022-00391-x.

## Introduction

Formal verification aims to guarantee the correctness of a given system with respect to a certain specification. However, the verification process might not be error-free and return incorrect results, even in well-known systems such as Mathematica [15]. In order to guarantee the correctness of the outcome, one would have to formally verify the verification tool, e.g., using a theorem prover, which typically is a demanding task and for complex software it is often infeasible. Thus, a more common technique to increase the trust in verification results is to generate proof certificates, which monitor steps of the verification process and enables reproducing the proof. These certificates can be checked by a simple stand-alone proof checker.

For example, many applications of formal verification use satisfiability (SAT) solving and various resolution or clausal proof formats [20], such as DRUP [57, 58], DRAT [22], and LRAT [14] are available to validate the verification results. In the annual SAT competition it is even required to provide certificates since 2013. However, in certain applications SAT solving cannot be applied successfully. For instance formal verification of arithmetic circuits, more precisely of multiplier circuits, is considered to be hard for SAT solving.

Automated reasoning based on computer algebra has a long history [27–29] with renewed recent interest. The general idea of this approach is to reformulate a problem as a question about sets of multivariate polynomials, then do Gröbner bases [8] computations and use properties of Gröbner bases to answer the question.

Formal verification using computer algebra provides one of the state-of-the-art techniques in verifying gate-level multipliers [11, 34, 46, 47]. In this approach the circuit is modeled as a set of polynomials and it is shown that the specification, also encoded as a polynomial, is implied by the polynomials that are induced by the circuit. More precisely, for each logical gate in the circuit a polynomial equation is defined that captures the relations of the inputs and output of the gate. The polynomials are sorted according to a term ordering that is consistent with the topological order of the circuit. This has the effect that these gate polynomials automatically generate a Gröbner basis [8]. Preprocessing techniques based on variable elimination are applied to simplify the representation of the Gröbner basis [34, 46]. After preprocessing the specification polynomial is reduced by the simplified gate polynomials using a multivariate polynomial division with remainder until no further reduction is possible. The given multiplier is correct if and only if the final result is zero.

Furthermore, algebraic reasoning in combination with SAT is successfully used to solve complex combinatorial problems [7], e.g., finding faster ways for matrix multiplication [23, 24], computing small unit-distance graphs with chromatic number 5 [19], or solving the Williamson conjecture [6], and has possible future applications in cryptanalysis [10, 56]. All these applications raise the need to invoke algebraic proof systems for proof validation.

Two algebraic proof systems are commonly studied in the proof complexity community, polynomial calculus (PC) [12], and Nullstellensatz (NSS) [3]. Both systems allow reasoning over polynomial equations where the variables represent Boolean values. These proof systems are well-studied, with the main focus on deriving complexity measures, such as degree and proof size, e.g., [2, 26, 49, 50].

Proofs in PC allow us to dynamically capture whether a polynomial can be derived from a given set of polynomials using algebraic ideal theory. However, PC as originally defined [12] is not suitable for effective proof checking [31], because information of the origin of the proof steps is missing. We introduce the practical algebraic calculus (PAC) [54], which includes this information and therefore can be checked efficiently. A proof in PAC is a sequence of proof steps, which model single polynomial operations. During proof checking each proof step is checked for correctness. Thus, whenever the proof contains an error, we are able to pinpoint the incorrect proof step.

In the first version of PAC [54] we explicitly require to write down all polynomial equations, including exponents, which leads to very large proof files. Since in our application all variables represent elements of the Boolean domain, we can impose for each variable *x* the equation $x^2 = x$. We use this observation and specialize PAC to treat exponents implicitly. That is, we immediately reduce all exponents greater than one in the polynomial calculations. Furthermore, we add an indexing scheme to PAC to address polynomial equations and add deletion rules for efficiency. We include a formalization of extension rules that allow us to merge and check combined proofs obtained from SAT and computer algebra [35] in a uniform (and now precise) manner (Sect. 2).

Proofs in NSS capture whether a polynomial can be represented as a linear combination of a given set of polynomials. These proofs are very concise as they consist only of the input polynomials and the sequence of corresponding co-factor polynomials. However, if the resulting polynomial is not equal to the desired target polynomial, it is unclear how to locate the error in the proof. Furthermore, it is impossible to express intermediate optimizations and rewriting techniques on the given set of polynomials in NSS, because we are not able to explicitly model preprocessing steps. We conjectured for the application of multiplier circuit verification [31] that: “In a correct NSS proof we would also need to express the rewritten polynomials as a linear combination of the given set of polynomials and thus loose the optimized representation, which will most likely lead to an exponential blow-up of monomials in the NSS proof.” Surprisingly, we have to reject our conjecture, at least for those multiplier architectures that are considered in our approach and our experimental results demonstrate that we are able to generate compact NSS proofs.

In this article we introduce LPAC, a PAC format including linear combinations that combines PAC with the strength of NSS (Sect. 3), namely a shorter proof, while retaining the possibility to identify errors. All proof formats can be produced by our verification tool AMulet 2.0 [33]. Depending on the options the proofs will have a stronger PAC, a hybrid, or a stronger NSS flavor (Sect. 4).

We present our new proof checkers Pacheck and Pastèque. They support PAC (Sect. 5). The proof checker Pastèque in contrast to Pacheck is verified in Isabelle/HOL, but Pacheck is faster and more memory efficient. To (in)validate our conjecture, we also implemented an NSS checker, Nuss-Checker. This gives us the evidence that NSS proofs do not lead to an exponential blow-up (Sect. 6). Therefore, we also extend Pacheck and Pastèque to check LPAC proofs (Sect. 7).

The tools are easy to use and their results can easily be interpreted. We experiment with the verification of various multipliers that require our new extensions to be checked. The new PAC format makes the proofs easier to check and less memory hungry, but proofs in LPAC achieve even better performance for both checkers (Sect. 8).

This article extends and revises work presented earlier [32, 39, 54]. As a novelty we introduce LPAC, the modification of the PAC format [39] to additionally support linear combinations of polynomials in the proof rules. Hence, we are able to not only simulate NSS and PC proofs in PAC, but we are also able to derive hybrid proofs that consist of a sequence of linear combinations. The hybrid format allows us to generate concise proofs, which are faster to check by our new checkers (Sect. 8), and where errors in the proof can be located. We present how LPAC proofs on different abstraction levels, i.e., NSS, hybrid or PC, are generated in our recent verification tool AMulet 2.0 [33]. Extending [39], we highlight necessary modifications in our proof checkers Pacheck and Pastèque to cover LPAC.

## Algebraic proof systems

In this section we introduce the proof systems polynomial calculus (PC) [12] and its instantiation PAC (Sect. 2.1) and Nullstellensatz [3] (Sect. 2.2). Our algebraic setting follows [13] and we assume $0 \in \mathbb N$.

- Let *R* be a ring and *X* denote the set of variables $\{x_1,\dots ,x_l\}$. By *R*[*X*] we denote the ring of polynomials in variables *X* with coefficients in *R*.
- A *term* $\tau =x_1^{d_1}\cdots x_l^{d_l}$ is a product of powers of variables for $d_i\in \mathbb N$. A *monomial* is a multiple of a term $c\tau$ with $c \in R\setminus \{0\}$ and a *polynomial* is a finite sum of monomials with pairwise distinct terms.
- On the set of terms [*X*] an order $\le$ is fixed such that for all terms $\tau ,\sigma _1,\sigma _2$ it holds that $1\le \tau$ and further $\sigma _1\le \sigma _2\Rightarrow \tau \sigma _1\le \tau \sigma _2$. One such order is the so-called *lexicographic term order*, defined as follows. If the variables of a polynomial are ordered $x_1> x_2> \dots > x_l$, then for any two distinct terms $\sigma _1= x_1^{d_1}\cdots x_l^{d_l}$, $\sigma _2= x_1^{e_1}\cdots x_l^{e_l}$ we have $\sigma _1 < \sigma _2$ iff there exists an index *i* with $d_j = e_j$ for all $j<i$, and $d_i<e_i$. We have $\sigma _1 = \sigma _2$ iff $d_j = e_j$ for all $1\le j \le l$.
- For a polynomial $p=c\tau + \cdots$ the largest term $\tau$ (w.r.t. $\le$) is called the *leading term* ${\text {lt}}(p)=\tau$. The *leading coefficient* ${\text {lc}}(p)=c$ and *leading monomial* ${\text {lm}}(p)=c\tau$ are defined accordingly. We call ${\text {tail}}(p)= p-{\text {lm}}(p)$ the *tail* of *p*.

As we will only consider polynomial equations with right hand side zero, we take the freedom to write *f* instead of $f = 0$. In our setting all variables represent Boolean variables, i.e., we are only interested in solutions where every variable $x \in X$ is assigned either 0 or 1. We can therefore impose the equations $x^2-x=0$ for all variables *x*. The set $B(X) = \{x^2-x \mid x \in X\} \subset R[X]$ is called the set of Boolean value constraints. Note that *R* is still an arbitrary ring as we do not restrict the coefficients of the polynomials, we only restrict the values of the variables.

### Definition 1

For a set $G\subseteq R[X]$, a *model* is a point $u=(u_1,\dots ,u_l)\in R^l$ such that $\forall g\in G : g(u)= g(u_1,\dots ,u_l)=0$. Here, by $g(u_1,\dots ,u_l)$ we mean the element of *R* obtained by evaluating the polynomial *g* for $x_1=u_1$, ..., $x_l=u_l$. Given $S \subseteq R$ a set $G\subseteq R[X]$ and a polynomial $f\in R[X]$, we write $G\models _{S} f$ if every model for *G* is also a model for $\{f\}$, i.e., $G\models _{S} f\iff \forall u \in S^l: \forall g\in G: g(u)=0\Rightarrow f(u)=0$.

Algebraic proof systems typically reason about polynomial equations. Given $G \subseteq R[X]$ and $f\in R[X]$, the aim is to show that an equation $f=0$ is implied by the constraints $g=0$ for every $g\in G\cup B(X)$. This means that every common Boolean root of the polynomials $g \in G$ is also a root of *f*. In algebraic terms, we want to derive whether *f* belongs to the ideal generated by $G \cup B(X)$.

### Definition 2

A nonempty subset $I \subseteq R[X]$ is called an *ideal* if $\forall u,v\in I: u+v\in I$ and $\forall w\in R[X],\forall u\in I: wu\in I$. If $G=\{g_1,\dots ,g_m\}\subseteq R[X]$, then the ideal generated by *G* is defined as $\langle G\rangle =\{q_1g_1+\dots +q_mg_m \mid q_1,\dots ,q_m\in R[X]\}$.

### Definition 3

Let $G \subseteq R[X]$ be a finite set of polynomials. A polynomial $f \in R[X]$ can be *deduced* from *G* if $f \in \langle {G} \rangle$. In this case we write $G\vdash f$.

### Polynomial calculus and PAC

The first proof system we consider is PC [12]. We discuss the original definition [12] over fields in Sect. 2.1.1 and generalize the soundness and completeness arguments. In Sect. 2.1.2 we generalize the correctness arguments to commutative rings with unity, when the constraint set *G* has a certain shape. For completeness the property “commutative ring with unity” is not sufficient and we will require stronger assumptions on the constraint set *G* in Sect. 2.1.2. In Sect. 2.1.3 we present our instantiation PAC.

#### Polynomial calculus over fields

In the original definition of PC [12] the coefficient ring *R* is assumed to be a field $\mathbb K$. Let $G\subseteq \mathbb K[X]$ and $f\in \mathbb K[X]$. A proof in PC is a sequence of polynomials $P = (p_1, \ldots , p_m)$ which are deduced by repeated application of the following proof rules:

$$\begin{aligned} \begin{array}{lll} \hbox {Addition }&{} \displaystyle \frac{p_i\qquad p_j}{p_i+p_j} &{}\begin{array}{l} p_i, p_j\hbox { appears earlier in the proof} \\ \hbox {or are contained in G}\end{array}\\ \\ \hbox {Multiplication }&{} \displaystyle \frac{p_i}{qp_i} &{} \begin{array}{l} p_i \hbox { appears earlier in the proof }\\ \hbox {or is contained in }\\ G \hbox { and }q\in \mathbb K[X] \hbox { being arbitrary}\end{array}\\ \end{array} \end{aligned}$$

We present here a variant of the PC where the addition and multiplication rules are closely related to the definition of an ideal. In the initial definition of PC [12], the addition rule is in fact a linear combination rule and includes multiplication by constants. The multiplication rule is more restrictive and only allows multiplication by a single variable $x \in X$ [12] or multiplication with any term, e.g., [9] instead of a polynomial. It is easy to see that our definition of PC and the original definition are equivalent and are able to simulate each other polynomially.

Note that every element $p_i$ of a PC proof *P* is an element of the ideal generated by *G*. This means that every common root of the elements of *G* is also a root of every polynomial appearing in the proof.

Thanks to the theory of Gröbner bases [4, 8, 13] the polynomial calculus is decidable, i.e., there is an algorithm which for any finite $G\subseteq \mathbb K[X]$ and $f\in \mathbb K[X]$ can decide whether $G\vdash f$ or not.

A basis of an ideal *I* is called a Gröbner basis if it enjoys certain structural properties whose precise definitions are not relevant for our purpose. What matters are the following fundamental facts:

- There is an algorithm (Buchberger’s algorithm) which for any given finite set $G\subseteq \mathbb K[X]$ computes a Gröbner basis *H* for the ideal $\langle G\rangle = \langle H\rangle$ generated by *G*.
- Given a Gröbner basis *H*, there is a computable function ${\text {red}}_H:\mathbb K[X]\rightarrow \mathbb K[X]$ such that $\forall \ p\in \mathbb K[X]:{\text {red}}_H(p)=0\iff p\in \langle H\rangle$.
- Moreover, if $H=\{h_1,\dots ,h_m\}$ is a Gröbner basis of an ideal *I* and $p,r\in \mathbb K[X]$ are such that ${\text {red}}_H(p)=r$, then there exist $q_1,\dots ,q_m\in \mathbb K[X]$ such that $p-r=q_1h_1+\cdots +q_mh_m$, and such co-factors $q_i$ can be computed.

In [12] soundness and completeness are shown for degree-bounded polynomials. In this context *soundness* means that every polynomial *f* which can be deduced by the rules of PC from a given set of polynomials *G* vanishes on every common root of the polynomials $g \in G$, i.e., $G \vdash f \implies G \models _{\mathbb K} f$. *Completeness* means whenever a polynomial *f* cannot be deduced by the rules of PC from *G*, then there exists a common root of the polynomials *G* where *f* does not evaluate to zero, i.e., $G \nvdash f \implies G \not \models _{\mathbb K} f$, or equivalently $G \models _{\mathbb K} f \implies G \vdash f$. We are able to generalize these arguments in this article without forcing a bound on the degree of *f* and the polynomials in *G*. At the end of this section we summarize how the results fit together in the context of algebraic verification.

To show soundness and completeness of PC over fields $\mathbb K$, we now introduce the extended calculus with the additional radical rule [13, Chap. 4§2 Def 2].

$$\begin{aligned} \begin{array}{lll} \hbox {Radical }&{} \displaystyle \frac{p^m}{p} &{} m\in \mathbb N\setminus \{0\} \hbox { and} p^m \hbox { appears earlier in the proof or is contained in }G.\\ \end{array} \end{aligned}$$

##### Definition 4

If the polynomial *f* can be deduced from the polynomials in *G* with the rules of PC and this additional radical rule, we write $G\vdash ^+ f$ and call this proof *radical proof*. In algebra, the set $\{\,f\in \mathbb K[X]:G\vdash ^+f\,\}$ is called the *radical ideal* of *G* and is typically denoted by $\sqrt{\langle G\rangle }$.

##### Theorem 1

Let $\mathbb K$ be an algebraically closed field and $G\subseteq \mathbb K[X]$, $f \in \mathbb K[X]$. It holds

$$\begin{aligned} G\vdash ^+f \iff G\models _{\mathbb K} f. \end{aligned}$$

##### Proof

It follows from Hilbert’s Nullstellensatz [13, Chap. 4§1 Thms. 1 and 2] that the set of all models of *G* is nonempty if and only if $1\not \in \langle G\rangle$, and furthermore we have $G\vdash ^+f\iff G\models _{\mathbb K} f$. $\square$

We are able to derive from Theorem 1 that the extended PC including the radical rule is correct (“$\Rightarrow$”) and complete (“$\Leftarrow$”).

Also the extended calculus $\vdash ^+$ is decidable. It can be reduced to $\vdash$ using the so-called Rabinowitsch trick [13, Chap. 4§2 Prop. 8], which says

$$\begin{aligned} f\in \sqrt{\langle G\rangle }\iff 1\in \langle G\cup \{yf-1\}\rangle \qquad \text {or}\qquad G\vdash ^+f\iff G\cup \{yf-1\}\vdash 1, \end{aligned}$$

depending whether you prefer algebraic or logic notation. In both cases, *y* is a new variable and the ideal/theory on the right hand sides is understood as an ideal/theory of the extended ring $\mathbb K[X,y]$.

##### Corollary 1

Let $\mathbb K$ be an algebraically closed field and assume $G\subseteq \mathbb K[X]$, $f \in \mathbb K[X]$, and $y \notin X$. We have $G\cup \{yf-1\}\vdash 1 \iff G\models _{\mathbb K} f$.

The Rabinowitsch trick is therefore used to replace a radical proof ($\vdash ^+$) by a PC refutation and we can therefore decide the existence of models and furthermore produce certificates for the non-existence of models using only the basic version of PC. Thus, we do not have to consider the radical rule in practice.

In Theorem 1 we consider models $u\in \mathbb K^l$. For our applications, only models $u\in \{0,1\}^l = \mathbb B^l \subseteq \mathbb K^l$ matter. Using basic properties of ideals [13, Chap. 4§3 Thm. 4], it is easy to show for $G \subseteq \mathbb K[X]$, $f \in \mathbb K[X]$ that $G\models _{\mathbb B}f\iff G\cup B(X)\models _{\mathbb K} f$. Recall from Definition 1 that $G\models _{\mathbb B}f\iff \forall u \in \mathbb B^l: \forall g\in G: g(u)=0\Rightarrow f(u)=0$.

Furthermore, the equivalence $G\cup B(X)\vdash ^+f \iff G\cup B(X)\models _{\mathbb K} f$ holds even when $\mathbb K$ is not algebraically closed, because changing from $\mathbb K$ to its algebraic closure $\overline{\mathbb K}$ will not have any effect on the models in $\mathbb B^l$. Finally, let us remark that the finiteness of $\mathbb B^l$ also implies that $G\cup B(X)\vdash ^+ f\iff G\cup B(X)\vdash f$. This follows from Seidenberg’s lemma [4, Lemma 8.13] and generalizes Thm. 1 of [12].

##### Corollary 2

Let $G\subseteq \mathbb K[X]$, $f \in \mathbb K[X]$, for any field $\mathbb K$. Then the following holds: $G\cup B(X)\vdash f \iff G\models _{\mathbb B} f$.

Let us briefly put the results of this section into context on the use case of formal verification. In algebraic verification the set *G* denotes the initial constraint set, e.g., for verifying circuits *G* contains all polynomials induced by a given circuit. The polynomial *f* encodes the specification. The goal of verification is to derive, whether *f* is implied by *G*, meaning that all common roots of the polynomials in *G* are roots of *f*, i.e. $G \models _{\mathbb K} f$. From $G \vdash f$ it trivially follows that $G \models _{\mathbb K} f$. However, the other direction $G \nvdash f \implies G \not \models _{\mathbb K} f$ does not hold in general. From Hilbert’s Nullstellensatz, cf. Theorem 1, we are only able to derive that $G \nvdash ^+ f \implies G \not \models _{\mathbb K} f$.

This means that in general an ideal membership test is not sufficient for verification and we would need to involve the stronger radical membership test to prove non-existence of models. Using the Rabinowitsch trick, cf. Corollary 1, allows us to replace the radical proof by an ideal membership test.

If all variables are Boolean, which is often the case in algebraic verification, we can further simplify Theorem 1, cf. Corollary 2. First, we relax on $\mathbb K$ being algebraically closed, because we are only considering a finite number of models $\mathbb B^l$. Second, because of the finiteness of $\mathbb B^l$, $G \cup B(X)$ is a zero-dimensional ideal, and using Seidenbergs’s Lemma we are able to deduce $\langle {G \cup B(X)} \rangle = \sqrt{\langle {G \cup B(X)} \rangle }$. Thus, we are able to replace the radical proof in Theorem 1 by an ideal membership test.

**Fig. 1:**
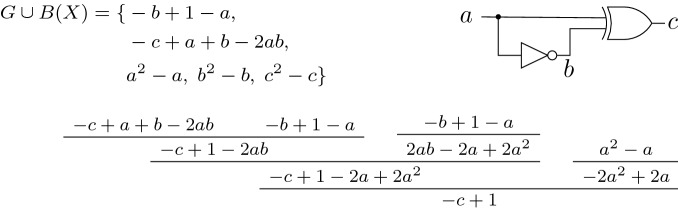
The circuit, polynomial representation of the gates and proof for Example 1

##### Example 1

This example shows that the output *c* of an XOR gate over an input *a* and its negation $b=\lnot a$ is always true, i.e., $c = 1$ or equivalently $-c + 1 =0$. We apply the polynomial calculus over the ring $R[X] = \mathbb K [X] = {\mathbb {Q}}[c,b,a]$. Over ${\mathbb {Q}}$ a NOT gate $x = \lnot y$ is modeled by the polynomial $-x+ 1-y$ and an XOR gate $z=x\oplus y$ is modeled by the polynomial $-z+x+y-2xy$. Because $X = \{a,b,c\}$, we have $B(X)=\{a^2-a, b^2-b, c^2-c\}$. The corresponding circuit representation, the constraint set $G \cup B(X)$, and a polynomial proof tree are shown in Fig. 1.

#### Polynomial calculus over commutative rings with unity

For certain sets of polynomials *G* we are further able to generalize the soundness and completeness arguments for rings *R*, which not necessarily have to be fields, e.g., $R = \mathbb Z$. Let now *R* denote a commutative ring with unity. By $R^\times$ we denote the set of multiplicatively invertible elements of *R*. The rules of PC remain unaffected.

##### Definition 5

Let $G \subseteq R[X]$. If for a certain term order, all leading terms of *G* only consist of a single variable with exponent 1 and are unique and further ${\text {lc}}(g) \in R^\times$ for all $g \in G$, then we say *G* has *unique monic leading terms* (UMLT). Let $X_0(G) \subseteq X$ be the set of all variables that do not occur as leading terms in *G*.

##### Example 2

The set $G=\{-x+2y, y-z\} \subseteq \mathbb Z [x,y,z]$ has UMLT for the lexicographic term order $x> y > z$. In this case $X_0(G)=\{z\}$.

##### Definition 6

Let $\varphi :X \rightarrow \mathbb B \subseteq R$ denote an *assignment* of all variables *X*. We extend $\varphi$ to an evaluation of polynomials in the natural way, i.e., $\varphi :R[X] \rightarrow R$.

##### Theorem 2

(Soundness) Let $G\subseteq R[X]$ be a finite set of polynomials and $f\in R[X]$, then

$$\begin{aligned} G\cup B(X)\vdash f ~~\Rightarrow ~~ G\models _{\mathbb B} f. \end{aligned}$$

##### Proof

If $G\cup B(X)\vdash f$ then $f\in \langle G\rangle +\langle {B(X)} \rangle$ by definition. This means there are $u_1,\dots ,u_m \in R[X]$ and $v_1,\dots ,v_r\in R[X]$ with $f=u_1g_1+\cdots +u_mg_m+v_1b_1+\cdots +v_rb_r$, where $g_i \in G$ and $b_i=x_i(x_i-1) \in B(X)$ for $i=1\ldots r$. Any assignment $\varphi$ in the sense of Definition 6 vanishes on *B*(*X*), i.e., $\varphi (b_i) = 0$. If $\varphi$ is also a model of *G* then $\varphi (g_i) = 0$ too and as a consequence $\varphi (f)=0$. Therefore $G\models _{\mathbb B} f$, as claimed. $\square$

Completeness is less obvious. Consider for instance that $\{2x\}\models _{\mathbb B} x$ but $x \not \in \langle 2x \rangle$ in $\mathbb Z[X]$. Requiring *G* to have UMLT turns out to be essential (which $\{ 2x \}$ does not have in $\mathbb Z[X]$, because $2 \notin \mathbb Z^\times$). Additionally, we will require the considered ring *R* to be an *integral domain*, which satisfies the property that the product of any two nonzero elements is nonzero [13].

##### Lemma 1

If $G \models _{\mathbb B} p$ and $G \models _{\mathbb B} q$ then $G \models _{\mathbb B} q \pm p$.

##### Lemma 2

Let $G \subseteq R[X]$ be a finite set of polynomials with UMLT. Then for all $q \in R[X]$ there exist $p \in \langle G\rangle + \langle {B(X)} \rangle$ and $r \in R[X_0(G)]$ with $q = p + r$, such that the variables in the monomials in *r* have only exponents 1.

##### Proof

We construct *p* and *r* by division of *q* by the polynomials in $G \cup B(X)$ until no term in *r* is divisible by any leading term of $G \cup B(X)$. First, we reduce *q* by the polynomials of *G*. Let $g_1 \in G$. Using polynomial division we are able to calculate $f_1, r_1 \in R[X]$ such that $q = f_1g_1 + r_1$ and no term in $r_1$ is a multiple of the leading term of $g_1$. We continuously divide the remainder by polynomials of *G* and derive $q = f_1g_1 + \cdots + f_mg_m + r_m$ for $g_i \in G$, $f_i, r_m \in R[X]$.

This process has to terminate because the tail of a polynomial contains only smaller variables and the number of variables in *G* is finite. Since *G* has UMLT, $r_m$ contains only variables in $X_0(G)$ which do not occur as leading terms, i.e, $r_m \in R[X_0(G)]$. If any of these variables occurs with exponent larger than one we can use *B*(*X*) to reduce their exponent to 1. Hence, we are able to derive $q = f_1g_1 + \cdots + f_mg_m +v_1b_1+\cdots +v_lb_l + r$, where $g_i \in G$, $b_i \in B(X)$, and $f_i, v_i \in R[X]$ and define $p = f_1g_1 + \cdots +f_mg_m +v_1b_1+\cdots +v_lb_l$. $\square$

##### Example 3

Let $G \subseteq \mathbb Z[x,y,z]$ be as in Example 2 and assume $q=2x^2+xy+z^2 \in \mathbb Z[x,y,z]$. Consequently

$$\begin{aligned} p&=(-2x{-}5y)(-x{+}2y)+(10y{+}10z)(y-z)-11(-z^2{+}z) \\&= 2x^2+xy+z^2-11z ~\in ~\langle G\rangle + \langle {B(X)} \rangle \text { ~and} \end{aligned}$$

$$r=11z ~\in ~\mathbb Z[X_0(G)]$$.

##### Lemma 3

Assume that *R* is an integral domain. Let $p \in R[X]$ with $p^2 - p \in \langle {B(X)} \rangle = \langle {\{ x^2 -x \mid x \in X \}} \rangle$. Further let $\varphi$ be an assignment in the sense of Definition 6. Then $\varphi (p) \in \mathbb B =\{0,1\}$.

##### Proof

Since $p^2 - p \in \langle {B(X)} \rangle$ there are $f_i \in R[X]$ with $p^2-p = \sum _i f_i\cdot (x_i^2-x_i)$. Thus, $\varphi (p^2-p) = 0$, as $\varphi$ vanishes on *B*(*X*). Assume now $\varphi (p) = \epsilon$ with $\epsilon \in R$. Then $\varphi (p^2-p) = \varphi (p)^2 - \varphi (p) = \epsilon ^2 - \epsilon = \epsilon (\epsilon - 1)$. As *R* is an integral domain, only $\epsilon \in \mathbb B$ yields $\varphi (p^2-p) = 0$. $\square$

##### Theorem 3

(Completeness) Let *R* be an integral domain and let $G \subseteq R[X]$ be a finite set of polynomials with UMLT. Suppose further that

$$\begin{aligned} \forall g \in G: ({\text {lc}}(g)^{-1}{\text {tail}}(g))^2 + ({\text {lc}}(g)^{-1}{\text {tail}}(g)) \in \langle {B(X)} \rangle . \end{aligned}$$

Then for every $f\in R[X]$ we have

$$\begin{aligned} G \models _{\mathbb B} f \quad \Rightarrow \quad G\cup B(X)\vdash f. \end{aligned}$$

##### Proof

Suppose we have $G \models _{\mathbb B} f$. Then our goal is to show $f\in \langle G\rangle +\langle {B(X)} \rangle$. First, by applying Lemma 2, we obtain $p \in \langle G\rangle +\langle {B(X)} \rangle$ and $r \in R[X_0(G)]$ with $f = p + r$. Thus $G\cup B(X)\vdash p$ by definition. Using Theorem 2 we derive $G \models _{\mathbb B} p$ and accordingly $G \models _{\mathbb B} f - p = r$ by Lemma 1. Now assume $r\ne 0$ and let *m* be a monomial of *r* which contains the smallest number of variables. Consider the assignment $\varphi$ that maps $x\in X_0(G)$ to 1 if it appears in *m* and to 0 otherwise. Therefore $\varphi (r) \ne 0$ since the coefficient of *m* is unequal to 0. This assignment on $X_0(G)$ admits a unique extension to *X* which vanishes on *G*. First, we consider the polynomial $\alpha x+t \in G$, where $\alpha \in R^\times$ and $t = {\text {tail}}(g)$, with the smallest leading term *x*. For this polynomial all variables in *t* are already considered in $\varphi$. Since $\alpha x+t = 0 \Leftrightarrow x = -\alpha ^{-1}t$ and we require $({\text {lc}}(g)^{-1}{\text {tail}}(g))^2 + ({\text {lc}}(g)^{-1}{\text {tail}}(g)) = (\alpha ^{-1}t)^2 + (\alpha ^{-1}t) = (-\alpha ^{-1}t)^2 - (-\alpha ^{-1}t) \in \langle {B(X)} \rangle$, we have $\varphi (-\alpha ^{-1}t) \in \{0,1\}$ by Lemma 3. We extend the assignment $\varphi$ to *x* by choosing $\varphi (x) = \varphi (-\alpha ^{-1}t)$. We continue in this fashion until all leading terms of *G* are assigned. Since *G* has UMLT we are able to derive such an assignment $\varphi$, which contradicts $G \models _{\mathbb B} r$. Thus $r=0$ and $f = p + r \in \langle G\rangle +\langle {B(X)} \rangle$. $\square$

In an earlier version of the manuscript, as well as in the conference paper [34, Thm. 2], the assumptions “$\forall g \in G: ({\text {lc}}(g)^{-1}{\text {tail}}(g))^2 + ({\text {lc}}(g)^{-1}{\text {tail}}(g)) \in \langle {B(X)} \rangle$” and “*R* is an integral domain” were missing. We thank one of the referees for making us aware of these bugs. If any of the three assumptions of Theorem 3 is missing, the theorem is wrong, as can be seen in the following examples.

First, let $G=\{xyz+xy-x-y\} \subseteq \mathbb Z[x,y,z]$ and $f=x-y \in \mathbb Z[x,y,z]$. The ring $R = \mathbb Z$ is an integral domain and we have $(xy-x-y)^2+xy-x-y\in \langle B(X)\rangle$. However *G* does not have UMLT, because the leading term of $xyz+xy-x-y$ consists of more than one variable. We have $G\models _{{\mathbb {B}}}f$ with the models $(x,y,z)=(0,0,0)$, (0, 0, 1), and (1, 1, 1), but $G\cup B(X)\not \vdash f$ because $r=x-y$.

Next, consider $G=\{-x+2y\} \subseteq \mathbb Z[x,y]$ and $f=y \in \mathbb Z[x,y]$. The polynomials in *G* have UMLT and $\mathbb Z$ is an integral domain. However, for the polynomial $-x+2y$ we have $4y^2-2y\not \in \langle B(X)\rangle$. We have $G\models _{{\mathbb {B}}}f$ with the model $(x,y)=(0,0)$ but $G\cup B(X)\not \vdash f$ because $r=y$.

Finally, let $G=\{x+4y\} \subseteq \mathbb Z_{10}[x,y]$ and $f=y \in \mathbb Z_{10}[x,y]$. The polynomial in *G* has UMLT, and we have $(4y)^2+4y = 6y^2-6y \in \langle B(X)\rangle .$ However the ring $R = \mathbb Z_{10}$ is not an integral domain as $5\cdot 2 = 0$. We have $G\models _{{\mathbb {B}}}f$ with the model $(x,y)=(0,0)$, but $G\cup B(X)\not \vdash f$ because $r=y$.

Although the previous example shows that the assumption that *R* is an integral domain cannot simply be dropped from Theorem 3, it is somewhat stronger than necessary. What really enters through Lemma 3 into the proof of Theorem 3 is the assumption that *R* is a ring in which the formula $\forall \ x\in R: x(x-1)=0\Rightarrow x=0\vee x=1$ is true. This holds in every integral domain, but also in some rings that are not integral domains, for example in rings $\mathbb Z_{2^k}$ for $k>1$. In our use case of algebraic circuit verification, which we introduce in Sect. 4.1, we choose $R=\mathbb Z_{2^k}$ for $k \ge 1$ to admit modular reasoning [34]. In the following lemma, we use Hensel lifting to prove that the rings $\mathbb Z_{2^k}$ have the desired property.

##### Lemma 4

Let $k\in \mathbb N\setminus \{0\}$, let $\varphi$ be an assignment in the sense of Definition 6, and let $p \in \mathbb Z_{2^k}[X]$ be such that $p^2 - p \in \langle {B(X)} \rangle$. Then $\varphi (p) \in \mathbb B =\{0,1\}$.

##### Proof

Proof by induction over *k*. Base case $k=1$: For $k=1$ the ring $\mathbb Z_2$ is a field. Since every field is an integral domain the base case follows by Lemma 3.

Induction step $k \rightarrow k+1$: Assume $p \in \mathbb Z_{2^{k+1}}[X]$ with $\varphi (p^2-p) = 0$ mod $2^{k+1}$. Let now $\varphi (p) = \epsilon$ with $\epsilon \in \mathbb Z_{2^{k+1}}$. Since $\epsilon \in \{0, \ldots , 2^{k+1}-1\}$ we can write $\epsilon = 2^k\epsilon _1 + \epsilon _0$ for $\epsilon _1 \in \{0,1\}$, $\epsilon _0 \in \{0, \ldots , 2^k-1\}$:First, since $k \ge 1$, we have $2^{2k} = 0$ mod $2^{k+1}$. Second, it follows that $\epsilon _0(\epsilon _0-1) = 0$ mod $2^k$. Thus by the induction hypothesis we have $\epsilon _0 \in \{0,1\}$ and the equation above simplifies toHence $\varphi (p) = \epsilon = \epsilon _0 \in \{0,1\}$. $\square$

##### Corollary 3

(Completeness for $\mathbb Z_{2^k}$) Let $R = \mathbb Z_{2^k}$ for $k \ge 1$ and
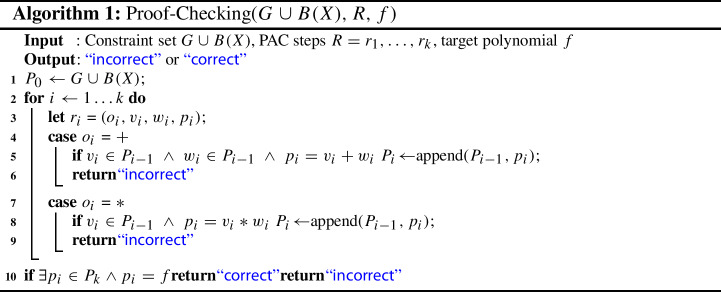
 let $G \subseteq R[X]$ be a finite set of polynomials with UMLT.

Suppose further that

$$\begin{aligned} \forall g \in G: ({\text {lc}}(g)^{-1}{\text {tail}}(g))^2 + ({\text {lc}}(g)^{-1}{\text {tail}}(g)) \in \langle {B(X)} \rangle . \end{aligned}$$

Then for every $f\in R[X]$ we have $G \models _{\mathbb B} f \Rightarrow G\cup B(X)\vdash f.$

In the use case of algebraic circuit verification, cf. Sect. 4.1, we automatically have “$\forall g \in G: ({\text {lc}}(g)^{-1}{\text {tail}}(g))^2 + ({\text {lc}}(g)^{-1}{\text {tail}}(g)) \in \langle {B(X)} \rangle$”. All polynomials $g \in G$ have the form $g:= -{\text {lt}}(g)+{\text {tail}}(g)$, with ${\text {lc}}(g) = -1$, and encode the relation between the output and inputs of a gate. The leading term ${\text {lt}}(g)$ represents the gate output and ${\text {tail}}(g)$ computes the output signal in terms of the inputs, cf., Fig. 3. Thus $\varphi ({\text {tail}}(g)) \in \{0,1\}$ and hence the assumption ${\text {tail}}(g)^2-{\text {tail}}(g) \in \langle {B(X)} \rangle$ holds.

#### Practical algebraic calculus

PC proofs as defined so far cannot be checked efficiently, because they only contain the conclusion polynomials of each proof step.

##### Example 4

Consider again the example of Fig. 1. The corresponding PC proof is $P = (-c+1-2ab, 2ab-2a+2a^2, -c+1-2a+2a^2, -2a^2+2a, -c+1)$. To check the correctness of this proof we would need to verify that each polynomial is derived using one of the PC rules, which is hard, because we do not have information on the antecedents.

For practical proof checking we translate the abstract rules of PC into a concrete proof format, i.e., we define a format based on PC, which is logically equivalent but more detailed. In principle a proof in PC can be seen as a finite sequence of polynomials derived from the initial constraint set and previously inferred polynomials by applying either an addition or multiplication rule. To ensure correctness of each proof step it is of course necessary to know which rule was used, to check that it was applied correctly, and in particular which given or previously derived polynomials are involved. During proof generation these polynomials are usually known and thus we require that all of this information is part of a rule in our concrete PAC proof format to simplify proof checking. A proof rule contains four components

$$\begin{aligned} o:v,w,p; \end{aligned}$$

The first component *o* denotes the operator which is either ‘+’ for addition or ‘*’ for multiplication. The next two components *v*, *w* specify the two (antecedent) polynomials used to derive *p* (conclusion). In the multiplication rule *w* plays the role of the polynomial *q* of the multiplication rule of PC.

For proof validation we need to make sure that two properties hold. The *connection property* states that the components *v*, *w* are either elements of the constraint set or conclusions of previously applied proof rules. For multiplication we only have to check this property for *v*, because *w* is an arbitrary polynomial. By the second property, called *inference property*, we verify the correctness of each proof step, namely we simply calculate $$v + w$$ resp. $$v * w $$ and check that the obtained result matches *p*. In a correct PAC proof we further need to verify that at least one conclusion polynomial *p* matches the target polynomial *f*. The complete checking algorithm is shown in Algorithm 1. Checking each step allows pinpointing the first error, instead of claiming that the proof is wrong somewhere in one of the (usually millions) steps.

##### Example 5

  Consider again the example presented in Example 1. One PAC proof obtaining $-c+1 \in \langle {G \cup B(X)} \rangle \subseteq \mathbb Q[X]$ is:
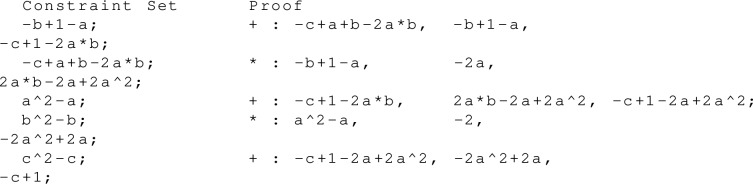


*Adaptions* We adapt PAC to admit shorter and more concise proofs. First, we index polynomials, i.e., each given polynomial and proof step is labeled by a unique positive number. It can be seen in Example 5 that the conclusion polynomial of the first proof step is again explicitly given as the first antecedent in the third proof step. Using indices, similar to LRAT [14], allows us now to label the first proof step and use this index in the third proof step. Naming polynomials by indices reduces the size of the proof files significantly and makes parsing more efficient, because only the conclusion polynomials of each step and the initial polynomials of *G* are stated explicitly. However, introducing indices for polynomials has the effect that the semantics changes from sets to multisets, as in DRAT [58], and it is possible to introduce the same polynomial under different names.

Second, we treat exponents implicitly. For bit-level verification [54] only models of the Boolean domain $\{0,1\}^n$ are of interest. Initially, we added the set of Boolean value constraints $B(X)=\{x^2-x \mid x \in X\}$ to *G* and have to include steps in the proofs that operate on these Boolean value constraints. Instead, we now handle operations on Boolean value constraints implicitly to reduce the number of proof steps. That is, we remove the Boolean value constraints from the constraint set and when checking the correctness, we immediately reduce exponents greater than one in the polynomials, i.e., $x^2 = x$.

Third, we further introduce a deletion rule to reduce the memory usage of the proof checker. After each proof step the conclusion polynomial will be added to the constraint set, thus the number of stored polynomial increases. If we know that a certain polynomial is not needed anymore in the proof, we use the deletion rule to remove polynomials.

We introduce the semantics of PAC as a transition system. Let *P* denote a sequence of polynomials which can be accessed via indices. We write $P(i)=\bot$ to denote that the sequence *P* at index *i* does not contain a polynomial, and $P(i \mapsto p)$ to denote that *P* at index *i* is set to *p*. The immediate reduction of exponents is denoted by “${\text {mod}}\langle B(X)\rangle$”. The initial state is $(X={\text {Var}}{\left( G \cup \{f\}\right) }, P)$ where *P* maps indices to polynomials of *G*. The following two rules implement the properties of ideals as introduced above for the original PAC.
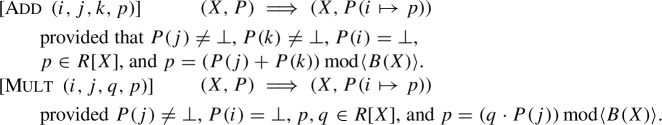
 In the deletion rule we remove polynomials from *P* which are not needed anymore in subsequent steps to reduce the memory usage of our tools.
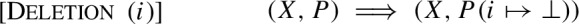


##### Example 6

  The proof of Example 1 in the adapted PAC format. We do not include all possible deletion steps in the proof.
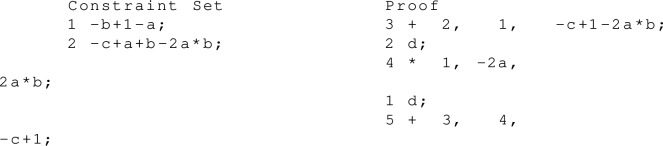


*Extension* Similar to the *polynomial calculus with resolution* (PCR) [1], which extends PC by a negation rule, we include an extension rule which allows us to add new polynomials to the constraint set. The negation rule of PCR introduces for each variable $x \in X$ an additional variable ${\overline{x}}$ that represents the negation of *x*. We generalize this extension rule such that new variables can act as placeholders for polynomials.

We use the extension rule to combine SAT solving and algebraic reasoning in our previous work [34] for multiplier verification. Thus, two proof certificates in different proof systems, DRUP and PAC are generated. In order to derive a single proof certificate we converted DRUP proofs to the PAC format [35]. However, to efficiently convert the resolution steps we encountered the need to extend the initial set of polynomials *G* to reduce the size of the polynomials (number of monomials) in the PAC proof. We included polynomials of the form $-f_x + 1 - x$, similar to the negation rule in PCR, which introduced the variable $f_x$ as the negation of the Boolean variable *x*. An example for modelling a resolution step in PAC is given in Example 7 below, where the proof step with index 3 demonstrates our new extension rule.

However, at that point we did not apply a proper extension rule, but simply added these extension polynomials to *G*. This may affect the models of the constraint set, because any arbitrary polynomial can be added as an initial constraint. For example, we could simply add the constant polynomial 1 to *G* which makes any PAC proof obsolete. To prevent this issue we add an extension rule to PAC, which allows us to add further polynomials to the knowledge base with new variables while preserving the original models on the original variable set of variables *X*.
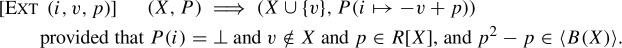


With this extension rule, variables *v* can act as placeholders for polynomials *p*, i.e., $-v+p=0$, which enables more concise proofs. The variables *v* are not allowed to occur earlier in the proof. Furthermore, to preserve Boolean models, we require $p^2-p\in \langle B(X)\rangle$. This can be easily checked by calculating $p^2-p$ and reducing all exponents larger than one to one. The normalized result has to be zero. Without this condition *v* might take non-Boolean solutions. In that case $v^n$ cannot be simplified to *v*, requiring to manipulate exponents in the proof checkers, which is currently not supported.

Consider for example $P=\{-y+x-1\}$. The only Boolean model is $(x,y)=(1,0)$. If we extend *G* by $-v+x+1$ we derive $v=2$, because $x=1$ for all models of *G*. Thus $v^2-v=0$ does not hold.

##### Proposition 1

Ext preserves the original models on *X*.

##### Proof

We show that adding $p_v:=-v+p$ does not affect the models of $G\cup B(X) \subseteq R[X]$. We have $\langle G\cup \{p_v\}\cup B(X \cup \{v\})\rangle = \langle G\cup \{p_v\}\cup B(X)\rangle$ because $v^2 - v = p^2 -p$ and $p^2-p\in \langle B(X)\rangle$. However, every model of $\langle G\cup \{p_v\}\cup B(X)\rangle$ is also a model of $\langle G\cup B(X)\rangle$ because the variable $v$ appears only as leading term in $p_v$. Hence the result. $\square$

The Isabelle formal proof is very similar to the idea given here, but we have to be more explicit. In particular, we explicitly manipulate a linear combination of the polynomials and show that every dependence in $v$ can be removed from the linear combination, since the variable $v$ appears only in $p_v$.

##### Example 7

Let ${\bar{x}} \vee {\bar{y}}$ and $y \vee z$ be two clauses. From these clauses we derive the clause ${\bar{x}} \vee z$ using resolution. The clauses are translated into polynomial equations using De Morgan’s laws and using the fact that a logical AND can be represented by multiplication. For example, from ${\bar{x}} \vee {\bar{y}} = \top \Leftrightarrow x\wedge y = \bot$ we derive the polynomial equation $xy=0$.

For the PAC proof we introduce an extension variable $f_z$, which models the negation of *z*, i.e. $-f_z+1-z =0$ in order to find a shorter representation of the second constraint, cf. proof step 5. 
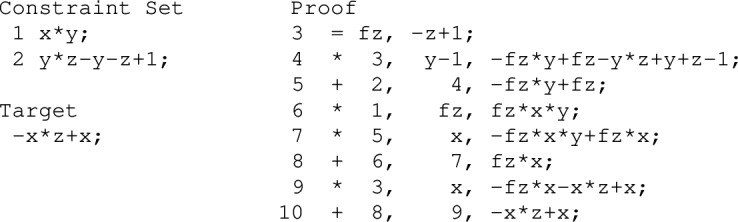


### Nullstellensatz

The *Nullstellensatz* (NSS) *proof system* [3] derives whether a polynomial $f \in R[X]$ can be represented as a linear combination of polynomials from a given set $G = \{g_1,\dots ,g_m\}\subseteq R[X]$. That is, an NSS proof for a given polynomial *f* and a set $G=\{g_1,\dots ,g_m\}$ is a tuple $P=(h_1,\dots ,h_m)$ of polynomials such that

$$\begin{aligned} \sum _{i=1}^{m}h_ig_i = f. \end{aligned}$$

By the same arguments given for PAC, the soundness and completeness arguments of NSS proofs can be generalized to rings *R*[*X*] when *G* has UMLT. In NSS the Boolean value constraints are treated implicitly to yield shorter proofs. Thus, the NSS proof we consider for a given polynomial $f \in R[X]$ and a set of polynomials $G = \{g_1,\dots ,g_m\}\subseteq R[X]$ is a tuple of co-factors $P=(h_1,\dots ,h_m)$ of polynomials such that there exist polynomials $r_1,\dots ,r_l \in R [X]$ with

1 $$\begin{aligned} \sum _{i=1}^{m}h_ig_i + \sum _{i=1}^l r_i (x_i^2-x_i) = f. \end{aligned}$$

Checking NSS proofs seems straightforward as we simply need to expand the products $h_ig_i$, calculate the sum, and compare the derived polynomial to the given target polynomial *f*. However, we discuss practical issues of proof checking in Sect. 6, where we introduce our NSS proof checker Nuss-Checker. Unlike PAC introduced above, NSS does not support extensions.

#### Example 8

A NSS proof for our running example introduced in Example 1 is 
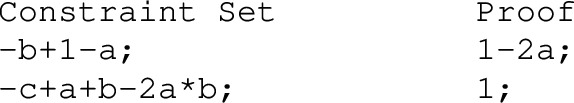


We derive $(1 - 2a)(-b+1-a) + (1)(-c+a+b-2ab) = -c+1~{\text {mod}}~ \langle B(X)\rangle$ in $\mathbb Q[X]$.

## Merging NSS and PAC into the hybrid proof system LPAC

PAC proofs are very fine-grained, because for each polynomial operation on the constraint set a single proof step is generated and checked for correctness. This makes it on the one hand simple to locate an error in the proof and thus to trace back the error in the automated reasoning tool. On the other hand the proof files are very large as for each proof step we write down a single line consisting of an index, the operation, two antecedents and the conclusion polynomial.

Nullstellensatz proofs are concise, as the core proof only consists of the ordered sequence of the co-factors, which has equal length of the constraint set. Thus the corresponding proof files are typically orders of magnitude smaller than PAC proofs, e.g., compare the proofs in Examples 6 and 8. However, because proof checking an NSS proof consists of calculating the linear combination and comparing it to the target polynomial, it is impossible to locate a possible error in the proof. Furthermore, the extensions of PAC are not directly portable to core NSS proofs.

**Fig. 2:**
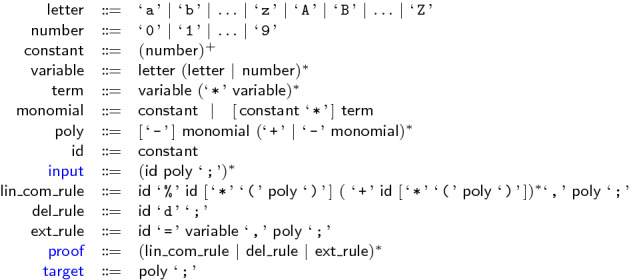
Syntax of input polynomials, target, and proofs in the LPAC-format

To take the best of both worlds we propose now a modified proof format, called LPAC (practical algebraic calculus + linear combinations). It includes a rule to merge the addition and multiplication rule to a single proof rule, which represents linear combination of polynomials. The syntax is given in Fig. 2. Thus we gain the following semantics. Let *P* denote a sequence of polynomials, which can be accessed via indices. The initial state is $(X={\text {Var}}{\left( G \cup \{f\}\right) }, P)$ where *P* maps indices to polynomials of *G*.
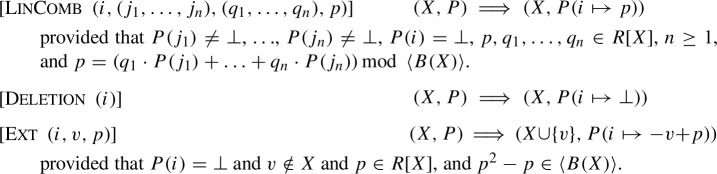


Our new LPAC format allows us to simulate both the PAC format and NSS proofs as follows. The LinComb is able to simulate both the Add and the Mult rule of PAC. By taking $n=2$, $(p_1, p_2)$, and (1, 1), we obtain the normal Add rule. By taking $n=1$, $(p_1)$, and $(q_1)$, we obtain Mult. The rules Deletion and Ext remain the same as for PAC. In the actual proof file, elements of the sequence $(q_1, \ldots , q_n)$ can be skipped and are interpreted as the constant sequence 1. We simulate NSS proofs by providing a single LinComb rule in the proof file.

Furthermore, we are able to generate hybrid proofs, which are not as concise as a single linear combination, but also not as fine-grained as an extended PAC proof. For example, in multiplier verification we apply polynomial reductions which always consist of a multiplication and addition of polynomials. In the LPAC proof format we are able to combine these two operations in a single proof step.

### Example 9

  A possible proof in LPAC for Example 1 is as follows:
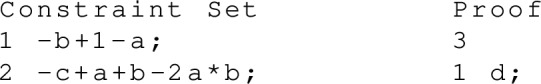


## Proof generation

In this section we demonstrate on the real-world application of multiplier verification how PAC, LPAC, and NSS proofs can be generated. We first provide a brief introduction to multiplier verification using our tool AMulet 2.0, before discussing how proof certificates can be generated.

### Multiplier verification

We developed a verification tool, called AMulet 2.0 [33, 34], which takes as input signed or unsigned integer multipliers *C*, given as And-Inverter-Graphs (AIGs), with 2*n* input bits $a_0,\ldots ,a_{n-1}$, $b_0,\ldots ,b_{n-1}\in \{0,1\}$ and output bits $s_0,\ldots ,s_{2n-1}\in \{0,1\}$. Nodes in the AIG represent logical conjunction and markings on the edges represent negation. We denote the internal AIG nodes by $l_1,\dots ,l_k\in \{0,1\}$. Let $\mathbb Z[X] =\mathbb Z[a_0,\dots ,a_{n-1},b_0,\dots ,b_{n-1},l_1,\dots ,l_k, s_0,\dots ,s_{2n-1}]$. In our application we require the coefficient domain to be $\mathbb Z$, because this allows us to apply modular reasoning by adding a constant $2^k$ to the set of ideal generators, which helps to keep the size of the intermediate verification results reasonably small. More details on modular reasoning are given in [34].

The multiplier *C* is correct iff for all possible inputs $a_i$, $b_i\in \{0,1\}$ the specification ${\mathcal {L}}=0$ holds:

2 $$\begin{aligned} {\mathcal {L}}= -\sum _{i=0}^{2n-1} 2^i s_i + \biggl (\sum _{i=0}^{n-1} 2^i a_i\biggr )\biggl (\sum _{i=0}^{n-1} 2^i b_i\biggr ) \end{aligned}$$

**Fig. 3:**
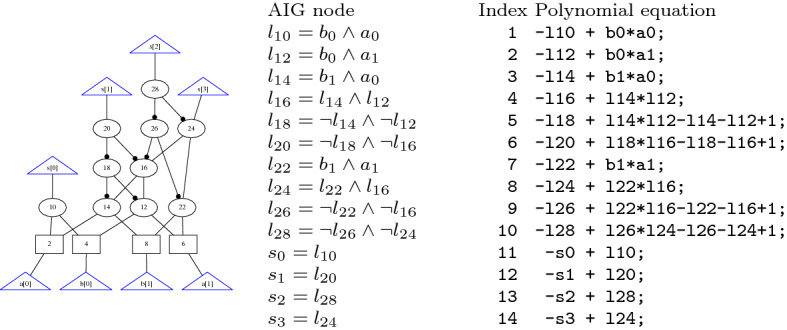
AIG of a simple 2 bit multiplier in AIGER format (left) with induced constraint set (right)

The semantics of each AIG node implies a polynomial relation, cf., Fig. 3. Let $G(C)\subseteq \mathbb Z[X]$ be the set of polynomials that contains for each AIG node of *C* the corresponding polynomial relation.

The polynomials in $G(C) \cup B(X)$ are ordered according to a lexicographic order, such that the output variable of a gate is always greater than the inputs of the gate, also called *reverse topological term order* (RTTO) [44]. Using this variable ordering leads to *G*(*C*) having UMLT.

Let $J(C) = \langle {G(C) \cup B(X)}\rangle \subseteq \mathbb Z[X]$ be the ideal generated by $G(C) \cup B(X)$. The circuit fulfills its specification if and only if we can derive that ${\mathcal {L}}\in J(C)$, which can be established by reducing ${\mathcal {L}}$ by the polynomials $G(C) \cup B(X)$ and checking whether the result is zero [34]. The algorithm for reducing a polynomial *p* by a second polynomial $p_v$ is shown in Algorithm 2. We again treat *B*(*X*) implicitly, thus we never explicitly reduce by a polynomial from *B*(*X*), but always cancel exponents greater than one to one, which is included in line 5. As a reduction order we follow the same order that is established for the variables.
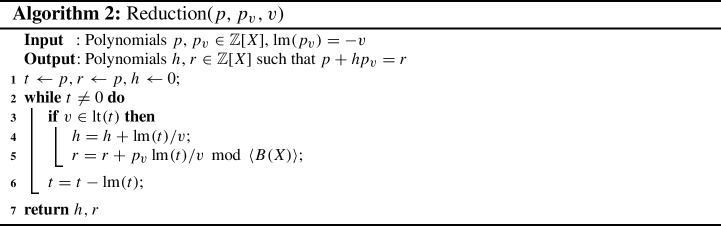


However, simply reducing the specification by *G*(*C*) leads to large intermediate results [45]. Hence, we eliminate variables in *G*(*C*) prior to reduction to yield a more compact polynomial representation of the circuit [34]. In the preprocessing step, we repeatedly eliminate selected variables $v \in X\setminus X_0$ from *G*(*C*), cf. Sect. 4.2. in [36]. Let $p_v \in G(C)$ such that ${\text {lt}}(p_v)=v$. Since *G*(*C*) has UMLT and $v \notin X_0$, such a $p_v$ exists. All polynomials *p*, with $v \in {\text {tail}}(p)$ are reduced by $p_v$ to remove *v* from *G* using Algorithm 2.

In contrast to more general polynomial division/reduction algorithms we use the fact in Algorithm 2 that ${\text {lm}}(p_v)=-v$. Because of the UMLT property and the fact that all leading coefficients of *G*(*C*) are -1, Algorithm 2 essentially boils down to substituting $v = {\text {lt}}(p_v)$ by ${\text {tail}}(p_v)$ in *p* in the case of circuit verification.

Algorithm 2 returns polynomials *h*, $r \in \mathbb Z[X]$ such that $p+hp_v = r ~{\text {mod}}~ \langle B(X)\rangle \in \mathbb Z[X]$. We replace the polynomial *p* by the calculated remainder *r* [34]. To keep track of the rewriting steps we want to store information on the derivation of the rewritten polynomial *r*.

### Generating PAC proofs

AMulet 2.0 generates PAC proofs as follows. The set of polynomials *G*(*C*) determines the initial constraint set. The specification ${\mathcal {L}}$ defines the target polynomial of the proof. Proof steps have to be generated whenever polynomials are manipulated, that is during preprocessing for variable elimination and during reduction.

For variable elimination we produce proof steps which simulate reduction of a polynomial *p* by a polynomial $p_v$, cf. Algorithm 2. Note that *p* and $p_v$ are both contained in *G*(*C*) and thus appear earlier in the proof. In general two proof steps are generated, a multiplication step and an addition step

$$\begin{aligned} id_i \texttt { * } h\texttt {,} id_{p_v}{} \texttt {,} hp_v\texttt {;} \qquad id_{i+1} \texttt { + } id_p\texttt {,} id_i\texttt {,} r\texttt {;} \end{aligned}$$

where $id_i$ and $id_{i+1}$ define unused indices, and $id_p$ and $id_{p_v}$ represent the indices of polynomials *p* resp. $p_v$. The polynomial $hp_v$ in above proof steps defines the expanded polynomial of multiplying $h \cdot p_v$ in $\mathbb Z[X]$. If ${\text {lt}}(p_v)=v$ does not occur in any other polynomial $g \in G(C)\setminus \{p_v\}$, we can delete $p_v$ from the constraint set, which we indicate by generating a deleting step

$$\begin{aligned} id_{p_v} ~\texttt {d;} \end{aligned}$$

After preprocessing is completed we gain the simplified polynomial model $G(C)'$. For monitoring the reduction of ${\mathcal {L}}$ by $G(C)'$ we have to generate proof steps which simulate the reduction of ${\mathcal {L}}$ by polynomials $g \in G(C)'$. We consider the polynomials $g \in G(C)'$ in the reverse topological order, such that each polynomial in $G(C)'$ has to be considered exactly once for reduction.

However in contrast to variable elimination, the specification ${\mathcal {L}}$, which acts as *p* in Algorithm 2, is not part of the constraint set. Thus we are not able to simply generate two proof steps as before, because checking the addition rule would raise an error, as $p = {\mathcal {L}}$ does not occur earlier in the proof. On the other hand recall that all elements of an ideal can be represented as a linear combination of the generators of the ideal. To simulate the linear combination we generate a multiplication PAC step for each reduction step by a polynomial $g \in G(C)'$ and store the computed factor *hg* (*h* is the returned co-factor of Algorithm 2). After reducing by several polynomials, we use a sequence of addition steps to gain a single intermediate specification polynomial. The reason for the intermediate summing up of polynomials is to keep the memory usage for proof generation small as we do not want to store too many factors at the same time. After reduction is completed we sum up all intermediate specifications. If the circuit is correct the final polynomial is the specification of the circuit.

#### Example 10

Figure 3 shows an AIG of a simple 2-bit multiplier. For each node we introduce the corresponding polynomial equation. These polynomials are shown on the right side of Fig. 3 and define the initial constraint set. The multiplier is correct if we derive that the gate polynomials imply the specification $-8s_3-4s_2-2s_1-s_0 + 4a_1b_1+2a_1b_0+2a_0b_1+a_0b_0=0$.

**Fig. 4:**
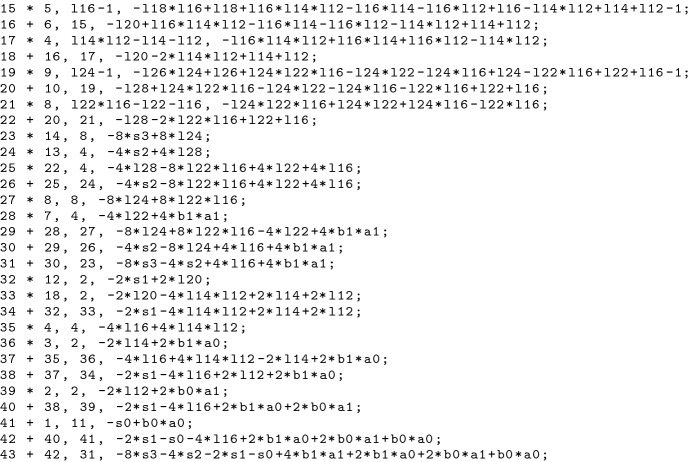
Generating PAC steps during multiplier verification


The corresponding PAC proof can be seen in Fig. 4. Steps 15–22 are generated during preprocessing. The remaining steps are generated during reduction of the specification by $G(C)'$. The result of step 43 matches the circuit specification.

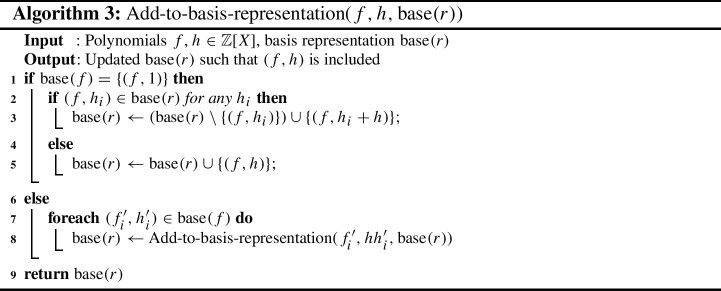


### Generating NSS proofs

In this section we discuss how NSS proofs are generated in our verification tool AMulet 2.0. We introduced in the previous section that we distinguish two phases during verification of multipliers. In the preprocessing step we eliminate variables from *G*(*C*) to gain a simpler polynomial representation $G(C)'$. In the second step the specification is reduced by $G(C)'$ to determine whether the given circuit is correct. Both phases have to be included in the NSS proof to yield a representation of the specification ${\mathcal {L}}$ as a linear combination of the original gate polynomials $G(C)\in \mathbb Z[X]$.

#### Definition 7

For a given set of polynomials $G \subset \mathbb Z[X]$, let ${\text {base}}(r)=\{(p_i, q_i) \mid p_i \in G$, $q_i \in \mathbb Z[X]\}$. We call ${\text {base}}(r)$ a basis representation of $r\in \mathbb Z[X]$ in terms of *G*, if there exist polynomials $v_1, \ldots v_l$ with $r = \sum _{(p_i,q_i)\; \in {\text {base}}(r)} q_ip_i + \sum _{i=1}^l v_i(x_i^2-x_i)$.

To derive a NSS proof for ${\mathcal {L}}$ we aim to find a basis representation of ${\mathcal {L}}$ in terms of *G*(*C*). For all polynomials $g \in G(C)$ it holds that ${\text {base}}(g) = \{(g,1)\}$ is a basis representation in terms of *G*(*C*).

As discussed in Sect. 4.1, we rewrite *G*(*C*) by replacing polynomials of *G*(*C*) by rewritten polynomials *r* that are derived using Algorithm 2. To keep track of the rewriting steps we store information on the derivation of the rewritten polynomial *r*, i.e., we derive a basis representation of *r* in terms of *G*(*C*). That is, we include the tuples (*p*, 1), $(p_v, h)$ as used in Algorithm 2 in ${\text {base}}(r)$.

Algorithm 3 shows how we update ${\text {base}}(r)$ by adding a tuple (*f*, *h*). If the input polynomial *f* of Algorithm 3 is an element of *G*(*C*), i.e. ${\text {base}}(f) = \{(f,1)\}$, we add the tuple (*f*, *h*) to ${\text {base}}(r)$. If *f* does not occur in any tuple in ${\text {base}}(r)$, we simply add (*f*, *h*) to ${\text {base}}(r)$. Otherwise ${\text {base}}(r)$ contains a tuple $(f, h_i)$ that has to be updated to $(f, h_i+h)$, which corresponds to merging common factors in ${\text {base}}(r)$.

If the polynomial *f* is not an original gate polynomial, *f* can be written as a linear combination $f = h'_1f_1 + \dots + h'_lf_l$ for some original polynomials $f_i$ and $h'_i \in \mathbb Z[X]$. Thus the tuple (*f*, *h*) corresponds to $hf = hh'_1f_1 + \dots + hh'_lf_l$. We traverse through the tuples $(f_i, h'_i) \in {\text {base}}(f)$, multiply each of the co-factors $h'_i$ by *h* and add the corresponding tuple $(f_i, hh'_i)$ to ${\text {base}}(r)$.

Multiplying and expanding the product $hh_i$ may lead to an exponential blow-up in the size of the NSS proof as the following example shows.

#### Example 11

Consider OR-gates $y_0 = x_0 \vee x_1$, $y_1 = y_0 \vee x_2$, $\ldots$, $y_k = y_{k-1} \vee x_{k+1}$ represented by the set of polynomials $G=\{-y_0+x_0+x_1-x_0x_1, -y_1+y_0+x_2-y_0x_2,\dots , -y_k+y_{k-1}+x_{k+1}-y_{k-1}x_{k+1})\}\subseteq \mathbb Z[y_0,\dots y_k, x_0, \dots x_{k+1}]$. Assume we eliminate $y_1,\dots ,y_{k-1}$, yielding $y_k = x_0 \vee x_1 \vee \ldots \vee x_{k+1}$. The expanded polynomial representation of $y_k$ contains $2^{k+2}$ monomials.

These sequences of OR-gates are common in carry-lookahead adders, which occur in complex multiplier architectures. This lead to the conjecture [31], which we stated in the introduction of this article. However, our previous verification approach [34] to tackle complex multipliers also relies on SAT solving. We substitute complex final-stage adders in multipliers by simple ripple-carry adders that do not rely on large OR-gates. Thus this blow-up does not occur in our experiments with our implementation (Sect. 6) for arithmetic circuit verification.

#### Example 12

We demonstrate a sample run of Algorithm 3. Let $G(C) = \{p_1, p_2, p_3\} \subseteq \mathbb Z[X]$ and $x,y,z \in \mathbb Z[X]$. Assume $q_1 = p_1 + xp_2$, $q_2= p_3 + yp_2$, and their basis representations ${\text {base}}(q_1)=\{(p_1, 1), (p_2, x)\}$ and ${\text {base}}(q_2) = \{(p_2, y), (p_3, 1)\}$. Let $p = q_1 + zq_2$. We receive the basis representation of *p* in terms of *G*(*C*) by adding $(q_1, 1)$ and $(q_2, z)$ to ${\text {base}}(p)$.

$(q_1, 1)$: Since $q_1 \notin G(C)$, we add each tuple of ${\text {base}}(q_1)=\{(p_1, 1), (p_2, x)\}$ with co-factors multiplied by 1 to ${\text {base}}(p)$.

$(q_2, z)$: We consider ${\text {base}}(q_2) = \{(p_2, y), (p_3, 1)\}$ and add $(p_2, yz)$ and $(p_3, z)$ to ${\text {base}}(p)$. Since $p_3$ is not yet contained in the ancestors of *p*, we directly add $(p_3, z)$ to ${\text {base}}(p)$. The polynomial $p_2$ is already contained in ${\text {base}}(p)$, thus we add *yz* to the co-factor *x* of $p_2$ and we derive ${\text {base}}(p) =\{(p_1, 1), (p_2, x+yz), (p_3, z)\}$.

After preprocessing is completed, we repeatedly apply Algorithm 2 and reduce the specification polynomial ${\mathcal {L}}$ by $G(C)'$. We generate the final NSS proof by deriving a basis representation for ${\mathcal {L}}$. Therefore we add after each reduction step the tuple (*g*, *h*), where *h* is the corresponding co-factor of polynomial *g*, to ${\text {base}}({\mathcal {L}})$ using Algorithm 3. After the final reduction step, ${\text {base}}({\mathcal {L}})$ represents an NSS proof and is printed to a file.

**Fig. 5:**
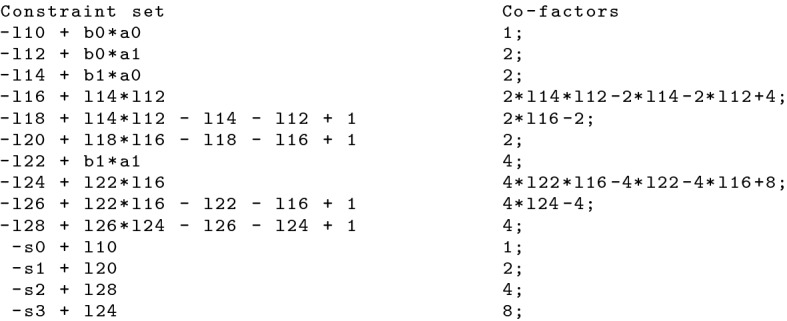
NSS proof for verifying the 2-bit multiplier that is depicted in Fig. 3

#### Example 13

Figure 5 shows the corresponding NSS proof for the verification of the 2-bit multiplier that is depicted in Fig. 3. It can be seen that the proof contains only the (ordered) co-factors and thus is smaller than the extensive PAC proof.

### Generating LPAC proofs

The LPAC format allows us to deliver dense PAC proofs. Thus, the proof generation is very similar as described in Sect. 4.2, with the difference being the level of compactness of the produced proof steps.

For each substitution step during preprocessing we generate a linear combination. That is, we merge the multiplication and addition steps, presented in Sect. 4.2 and gain for each preprocessing step a single step

$$\begin{aligned} id_i ~{\texttt {\%}} ~id_{p_v} \texttt {*(}h\texttt {) + }id_p\texttt {, }r\texttt {;} \end{aligned}$$

Similar as before, we generate deletion steps whenever $p_v$ can be removed.

During the reduction phase we calculate and store the factors of each reduction step. After reducing by several polynomials we generate a linear combination step which sums up these factors to gain intermediate specifications. Thus, we are able to narrow down possible errors. Finally, we sum up the intermediate specifications in a single step and yield the specification ${\mathcal {L}}$.

**Fig. 6:**
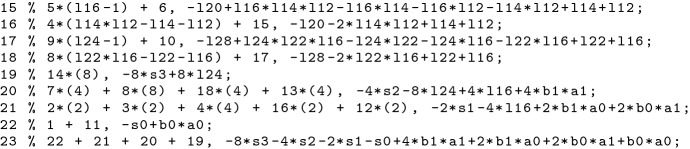
Generating a LPAC proof during multiplier verification

#### Example 14

Figure 6 shows the corresponding LPAC proof for the verification of the 2-bit multiplier that is depicted in Fig. 3. The proof steps 15–18 are generated during preprocessing, 19–22 are generated during reduction and step with index 23 is the final step for summing up the intermediate specifications. It can be seen that LPAC enables merging PAC steps. For example the steps with indices 15 and 16 of Fig. 4 are now combined in the first proof step.

## PAC checkers

We have implemented two checkers for PAC proofs. The first, Pacheck, (Sect. 5.1) is efficient while the second, Pastèque, is verified using Isabelle/HOL (Sect. 5.2).

### Pacheck 1.0

Pacheck consists of approximately 1 800 source lines of C code and is published [38] under MIT license. The default mode of Pacheck supports the extended version of PAC for the new syntax using indices. Pacheck also supports reasoning with exponents as described in the initial version of PAC. However, extension rules are only supported for Boolean models.

Pacheck reads three input files $\texttt {<constraints>}$, $\texttt {<proof>}$, and $\texttt {<target>}$ and then verifies that the polynomial in $\texttt {<target>}$ is contained in the ideal generated by the polynomials in $\texttt {<constraints>}$ using the proof steps provided in $\texttt {<proof>}$. The polynomial arithmetic needed for checking the proof steps is implemented from scratch, because in the default setting we always calculate modulo the ideal $\langle B(X)\rangle$. General algorithms for polynomial arithmetic need to take exponent arithmetic over $\mathbb Z$ into account [55], which is not the case in our setting.

**Fig. 7:**
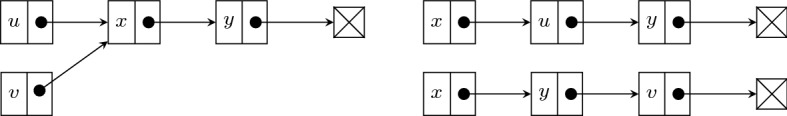
Term representation w.r.t. $v>u>x>y$ (left) and $x>u>y>v$ (right)

In the default mode of Pacheck we order variables in terms lexicographically using !strcmp!. All internally allocated terms are shared using a hash table. It turns out that the order of variables has an enormous effect on memory usage, since different variable orderings induce different terms. For example, given the monomials *uxy* and *vxy*. For the ordering $v>u>x>y$, the internal sharing is maximal and only 4 terms are allocated. For the ordering $x>u>y>v$, terms cannot be shared and thus 6 terms need to be allocated, cf. Figure 7. For one example with more than 7 million proof steps, using !-1*strcmp! as sorting function leads to an increase of 50% in memory usage. A further option for sorting the variables is to use the variable appearance ordering from the given proof files. That is, we assign increasing !level! values to new variables during parsing of the proof file and sort according to this value. However, the best ordering that maximizes internal sharing cannot be determined in advance from the original constraint set, as it highly depends on the applied operations in the proof steps. Pacheck supports the orderings !strcmp!, !-1*strcmp!, !level!, and !-1*level!. Terms in polynomials are sorted using a lexicographic term order that is induced by the order of the variables.

Initially each polynomial from $\texttt {<constraints>}$ is sorted and stored as an inference. Inferences consist of a given index and a polynomial and are stored in a hash table. Proof checking is applied on-the-fly. We parse each step of $\texttt {<proof>}$ and immediately apply the necessary checks discussed in Sect. 2.1.3. If the proof step is either Add or Mult , we have to compute whether the conclusion polynomial of the step is equal to the arithmetic operation performed on the antecedent polynomials.

Since the monomials of the polynomials are sorted, addition of polynomials is performed by merging their monomials in an interleaved way. Normalization of the exponents is not necessary in the Add rule, but we still use this technique for multiplication, where we multiply each monomial of the first polynomial with each monomial of the second polynomial. In the Mult rule we normalize exponents larger than one, before testing equality. Furthermore, we check whether the conclusion polynomial of the Add or Mult steps matches the polynomial in $\texttt {<target>}$ to identify whether the normalized target polynomial was derived.

### Pastèque 1.0

To further increase trust in the verification, we implemented a verified checker called Pastèque in the proof assistant Isabelle/HOL [52]. It follows a “refinement” approach, starting with an abstract specification of ideals, which we then refine with the Isabelle Refinement Framework [41] to the transition system from Sect. 2, and further down to executable code using Isabelle’s code generator [18]. The Isabelle files have been made available [17]. The generated code consists of 2 800 lines Standard ML (2 400 generated by Isabelle, 400 for the parser) and is also available [17, 38] under MIT license.

On the most abstract level, we start from Isabelle’s definition of ideals. The specification states that if “success” is returned, the target is in the ideal. Then we formalize PAC and prove that the generated ideal is not changed by the proof steps. Proving that PAC respects the specification on ideals was not obvious due to limited automation and development of the Isabelle library of polynomials (e.g., “${\text {Var}}{(1)} = \emptyset$” is not present). However, Sledgehammer [5] automatically proved many of these simple lemmas. We made a slightly different choice for definitions: Instead of using $B(X)=\{x^2-x\mid x\in X\}$, we used $\{x^2-x\mid \text {True}\}$ and proved that we only need variables of $X$. This made little difference for proofs, but avoided checking that variables are present in the problem.

While the input format identifies variables as strings, Isabelle only supports natural numbers as variables. Therefore, we use an injective function to convert between the abstract specification of polynomials (with natural numbers as variables) and the concrete manipulations (with strings as variables). The code does not depend on this function, only the correctness theorem does. Injectivity is only required to check that extension variables did not occur before.

In the third refinement stage, Sepref [40] changes data structures automatically, such as replacing the set of variables *X* by a hash-set. Finally, we use the code generator to produce code. This code is combined with a trusted (unproven) parser and can be compiled using the Standard ML compiler MLton [59].

The implementation does not support the usage of exponents and is less sophisticated than Pacheck ’s. In particular, even if terms are sorted, sharing is not considered (neither of variables or of monomials) as it can be executed partially by the compiler, although not guaranteed by Standard ML semantics. Some sharing could also be performed by the garbage collector. We tried to enforce sharing by using MLton’s shareAll function and by using a hash map during parsing, i.e., using a hash map that assigns a variable to “itself” (the same string, but potentially at a different memory location) and normalize every occurrence. However, performance became worse.

Pastèque is four times slower than Pacheck. First, this is due to Standard ML being intrinsically slower than C or C++. While Isabelle’s code generator to LLVM [43] produces much faster code, we need integers of arbitrary large size, which is currently not supported. Also achieving sharing is entirely manual, which is challenging due to the use of separation logic Sepref. Second, there is no axiomatization of file reading and hence parsing must be applied *entirely* before calling the checker in order for the correctness theorem to apply. This is more memory intensive and less efficient than interleaving parsing and checking. Pastèque can be configured via the uloop option to either use the main loop generated by Isabelle (parsing before calling the generated checker) or instead use a hand-written copy of the main loop, the *unsafe loop*, where parsing and checking is interleaved. It is only unsafe because it is unchecked. However, the performance gain is large (on sp-ar-cl-64 with ${32}\,\hbox {GB}$ RAM, the garbage collection time went from ${700}\,\mathrm{s}$ down to ${25}\,{\mathrm{s}}$), but only the checking functions of each step are verified, not the main loop.

## The NSS checker Nuss-Checker

Our NSS proof checker, Nuss-Checker is implemented in C. It consists of $\sim$ 1500 source lines of code and is published [30] as open source under the MIT license. Similar to Pacheck, Nuss-Checker reads three input files $\texttt {<constraints>}$, $\texttt {<cofact>}$, and $\texttt {<target>}$. The file $\texttt {<constraints>}$ contains the initial constraints $g_i \in G$, $\texttt {<cofact>}$ contains the corresponding co-factors $h_i$ in the same order. Nuss-Checker reads the files $\texttt {<constraints>}$ and $\texttt {<cofact>}$, generates the products and then verifies that the sum of the products is equal to the polynomial *f* given in $\texttt {<target>}$. Nuss-Checker uses the same internal representation of polynomials as Pacheck and furthermore supports the same variables orders as Pacheck, with !strcmp! being the default ordering.

We validate the correctness of the generated NSS proofs by checking whether $\sum _{i=1}^{l}h_ig_i = f \in \mathbb Z[X]$ for $p_i \in G \subseteq \mathbb Z[X]$, $f, h_i \in \mathbb Z[X]$. This sounds rather straightforward as theoretically we only need to multiply the original constraints $g_i$ by the co-factors $h_i$ and calculate the sum of the products. However, we will discuss in this section that depending on the implementation the time and maximum amount of memory that is allocated varies by orders of magnitude.

**Fig. 8:**
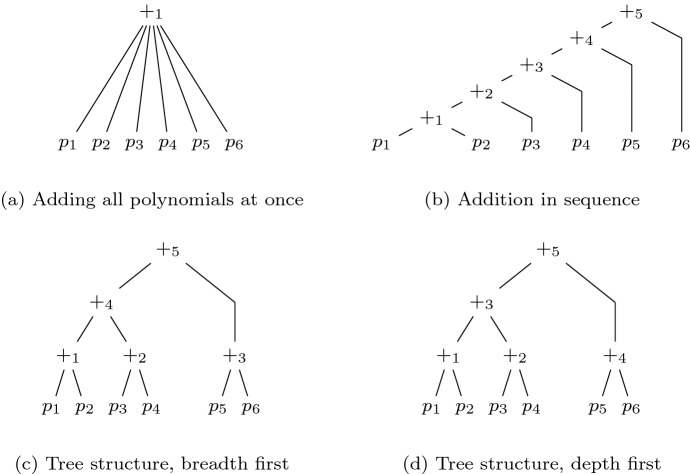
Addition schemes of 6 polynomials

Nuss-Checker generates the products $h_ig_i$ on the fly. That is, we parse both files $\texttt {<constraints>}$ and $\texttt {<cofact>}$ simultaneously, read two polynomials $g_i$ and $h_i$ from each file and calculate $h_ig_i$. Since addition of polynomials in $\mathbb Z[X]$ is associative, we are able to derive different addition schemes for n-ary addition. We experimented with five different addition/subtraction patterns. The addition patterns are depicted in Fig. 8 for adding six polynomials. The subscript *i* in “$+_i$” shows the order of the addition operation.

If we *sum up all polynomials at once*, we do not generate the intermediate addition results. Instead we push all monomials of the *l* products $h_ig_i$ onto one big stack. Afterwards, the monomials on the stack are sorted and merged, which corresponds to one big addition. However, all occurring monomials of the products are pushed on the stack and stored until the final sorting and merging, which increases the memory usage of Nuss-Checker.

If we *add up in sequence*, we only store one polynomial in the memory, and always add the latest product $h_ig_i$. On the one hand, this allows for monomials to cancel, which helps to reduce the memory usage. On the other hand, in the application of multiplier verification (cf. Sect. 4.1) the target polynomial ${\mathcal {L}}$ contains $n^2$ partial products $a_ib_j$ that lead to intermediate summands of quadratic size, which slows down the checking time.

For adding up in sequence we also experimented with the “inverse” operation, where we start with the target polynomial and step by step *subtract* the products $h_ig_i$ in the order originally used during the verification. We check whether the final polynomial is equal to zero. Again we always store only one polynomial in the memory, which admits a low memory usage. However, in our application the target polynomial is of quadratic size, making step-wise subtractions time-consuming.

If we add up in a *tree structure with breadth first*, we add two consecutive products of the NSS proof and store the resulting sum. After parsing the proof, we have $\frac{l}{2}$ polynomials on a stack. We repeatedly iterate over the stack and always sum up two consecutive polynomials, until only one polynomial is left. Using a tree addition scheme reduces the likelihood of quadratic sized intermediate summands for multiplier verification.

In the addition scheme, where we use a *tree structure and sum up depth first*, we develop the tree on-the-fly by always adding two polynomials of the same layer as soon as possible. It may be necessary to sum up remaining intermediate polynomials that are elements of different layers, as shown in Fig. 8. We always store at most $\lceil \log (l)\rceil$ polynomials in the memory, as a binary tree with *l* leafs has height $\lceil \log (l)\rceil$ and we never have more polynomials than layers in the memory.

**Fig. 9:**
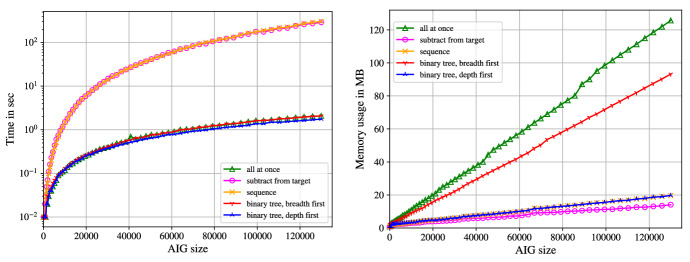
Time (left) and memory usage (right) of addition schemes for btor multipliers

**Fig. 10:**
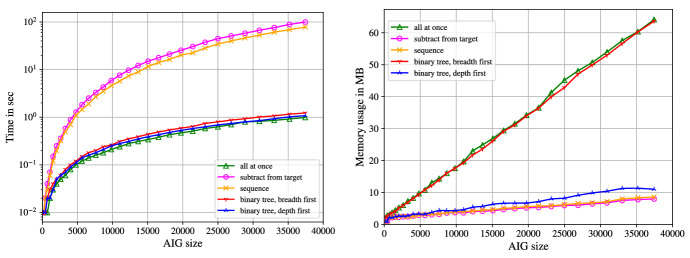
Time (left) and memory usage (right) of addition schemes for bp-wt-rc multipliers

We apply the presented addition schemes for our use case of multiplier verification. We choose two multiplier architectures. In our first experiment we consider a simple multiplier architecture, called *btor*, that is generated using Boolector [51] for various input sizes. Second, we examine a more complex multiplier architecture, called *bp-wt-rc*, that uses a Booth encoding and Wallace-tree accumulation. Figures 9 and 10 show that the results compare favorably to our conjectures of checking time and memory usage for each addition scheme. However, Nuss-Checker supports all presented options for addition, with *adding up in binary tree, depth first* set as default, because for different applications, using other addition schemes may be more beneficial. For example, we shuffled the order of the polynomials in the NSS proof of 128-bit btor-multipliers 200 times. The addition schemes “adding up in sequence” and “subtract” always exceeded the time limit of 300 seconds. The fastest addition scheme is “all at once”, which is a factor of two faster than both tree-based addition schemes.

## LPAC checkers

The LPAC checkers combine the strength of PAC (checking intermediate steps and supporting extensions), while allowing doing a linear combination in a single step like NSS proofs. We have extended Pacheck (Sect. 7.1), based on our experiments for Nuss-Checker, and Pastèque (Sect. 7.2) to Pacheck 2.0 and Pastèque 2.0.

### Pacheck 2

Pacheck 2.0 is a re-factorization and improved C++ reimplementation of our previous proof checkers. Since we are able to simulate PAC and NSS proofs in LPAC, Pacheck 2.0 unites and extends Pacheck 1.0 and Nuss-Checker.

**Fig. 11:**
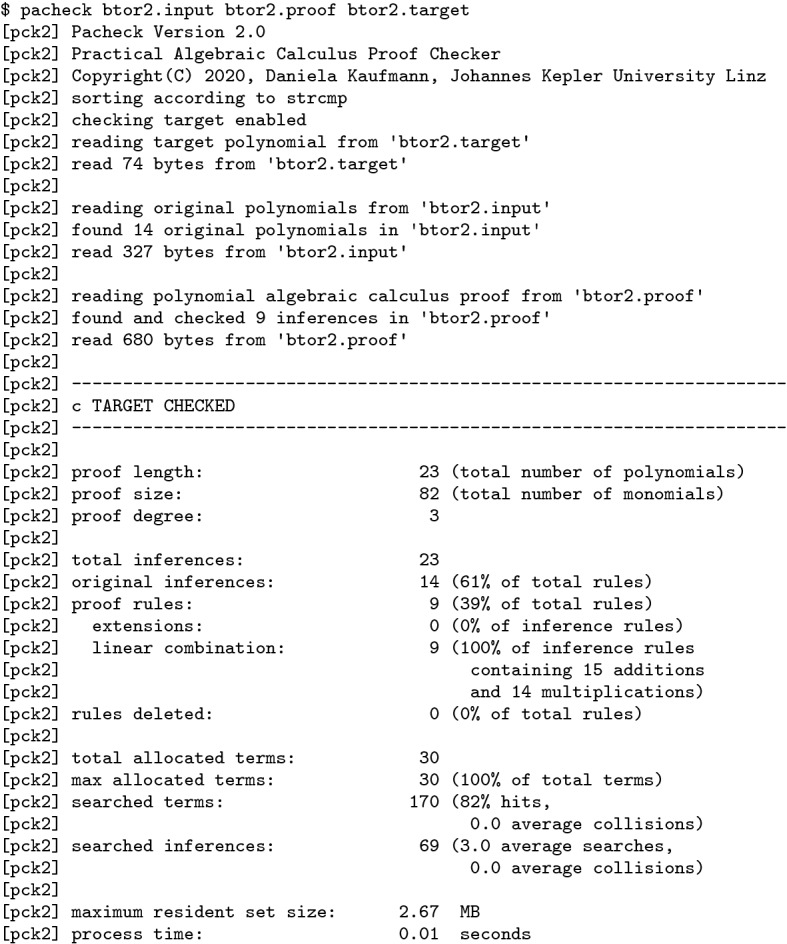
Output of Pacheck 2.0 for the proof of Example 14

The internal representation of polynomials is almost the same as for Pacheck 1.0. However, Pacheck 2.0 does no longer support the usage of exponents and thus only supports Boolean models. Proof checking is applied on the fly. That is, we parse a proof step and calculate that the linear combination of known polynomials is equal to the given conclusion polynomial of the proof step. We calculate linear combinations similar to proof checking a NSS proof in Nuss-Checker, i.e., whenever we parse a product of a polynomial and an index, we directly calculate the factor. The factors of the linear combination are processed using a *tree structure with depth first* addition scheme. Figure 11 shows a demonstration of Pacheck 2.0 on the LPAC proof of Example 14.

### Pastèque 2

Pastèque 2.0 [16] is developed on top of Pastèque 1.0. In order to reuse as much as possible from Pastèque 1.0, we reuse the specification and the rules of PAC. Instead of proving the correctness of the LPAC rules directly, we reduce them to the PAC rules, by seeing the LinComb rule as a series of Add and Mult . This requires the linear combination to not be empty: While 0 is always in the ideal, it cannot be generated by the PAC rules.

Additionally, we introduced explicit sharing of variables. We map every variable string to a unique 64-bit machine integer. In turn, this integer is the index of the original string in an array. Sharing is introduced in a new refinement step. The major change is that importing a new variable can now fail (if the problem contains more than $2^{64}$ different variables). This is nearly impossible in practical problems, but we had to add several new error paths in Pastèque. We obviously set up the code generator to make the array access from machine words in an array without converting it to an unbounded integer. This change give us a performance improvement of around $10\%$, most likely because the memory representation is more efficient (fewer pointer indirections), making the work of the garbage collector easier.

On top of that, as we know that all our array accesses are valid (this is checked by Sepref during synthesis of the code),^1^ we add a flag such the compiler makes use of that. This also allowed us to use MLton’s LLVM backend that produce faster code, according to our experiments.

We did not change the implementation of the uloop option. Like Pastèque 1.0, a full proof step is parsed before being checking. For NSS-style LPAC proof, this means that the full proof is still parsed before checking. In particular, for such proofs, Pastèque 2.0 should be compared the default version of Pastèque 1.0. The new sharing reduces memory usage, but parsing the full proof still causes a extreme memory pressure, as demonstrated by the experiments (Sect. 8). A solution would be to move the parsing to Isabelle (i.e., take a string as input instead of polynomials).

## Experiments

**Table 1:** Proof checking (in bold the fastest version)

Multiplier	2*n*	Steps	Pacheck 1.0						Pastèque 1.0			
			No delete		No index		Default		Default		uloop	
		($10^6$)	sec	MB	sec	MB	sec	MB	sec	MB	sec	MB
btor	128	0.3	**5**	273	11	100	**5**	92	22	3886	17	1773
btor	256	1.0	**25**	1144	62	435	**25**	364	105	21,157	79	4364
btor	512	4.2	**138**	4956	402	1972	141	1461	531	64,412	416	22,292
sp-ar-rc	128	0.4	**6**	454	16	148	**6**	136	31	5002	23	1608
sp-ar-rc	256	1.6	29	1858	96	651	**27**	541	139	32,525	102	8769
sp-ar-rc	512	6.3	146	7683	617	2965	**134**	2171	608	64,412	471	25,632
sp-ar-cl	32	1.6	23	773	36	354	**21**	353	121	40,654	113	9492
sp-dt-lf	32	0.3	**2**	122	3	73	**2**	73	11	1679	11	886
bp-ct-bk	32	0.2	**1**	86	2	52	**1**	51	8	1600	7	1 068
bp-wt-cl	32	5.6	193	4324	302	1430	**181**	1428	786	58,867	774	64,404

In our experiments we use an Intel Xeon E5-2620 v4 CPU at 2.10 GHz (with turbo-mode disabled) with a memory limit of 128 GB. The time is listed in rounded seconds (wall-clock time). We measure the wall-clock time from starting the tools until they are finished. In our experiments we aim to provide a comprehensive comparison between our tools. Source code, benchmarks and experimental data are available [37].

### PAC proofs

For the experiments of Table 1 we generate PAC proofs as in previous work [34, 35] to validate the correctness of multipliers with input bit-width *n*. The circuits are either generated with AMG [25], Boolector [51], or GenMul [48].

For the upper part of Table 1 we generate proof certificates with our tool AMulet 2.0 [33] to validate the correctness of simple multiplier circuits. Our previous approach [34] to tackle complex multipliers also relies on SAT solving. We substitute complex final-stage adders in multipliers by simple ripple-carry adders. A bit-level miter is generated, which is passed on to a SAT solver to verify the equivalence of the adders. Computer algebra techniques are used to verify the rewritten multiplier. Since two different solving techniques are used, two proof certificates in distinct formats are generated. SAT solvers generate a DRUP proof and computer algebra techniques produce a PAC proof. In order to obtain a single proof certificate we translate DRUP proofs into PAC [35]. In the experiments of [35] all gate polynomials of the given multiplier, the equivalent ripple-carry adder, and the bit-level miter are assumed as initial set of constraints *G*. We even added polynomials that define Boolean negation to the initial constraint set. All these polynomials are now added using extension steps. This preserves the models of the gate polynomials of the given multiplier. Experiments for these proof certificates are shown in the lower part of Table 1. The second column shows the input bit-width and the third column shows the number of generated proof steps.

The memory usage for Pastèque depends on the garbage collector, which likely explains the peak around 64 GB, that is exactly half of the available memory, observed for the largest problems. Details on when and how the garbage collection trigger could explain the surprising bp-wt-cl where the uloop option uses more memory.

The effect of deletion rules and indices in Pacheck can also be seen in Table 1. In average deletion rules reduce the memory usage by $\sim$60%, with minimum 40% (for bp-ct-bk) and maximum 72% (for sp-ar-rc 512). Although the effect on runtime is limited. Using indices reduces the runtime by 30 to 80%. Note that in our earlier experiments [35] the proof checking time is slightly faster than in the column “no index”, because we did not use proper extension rules, which requires the additional checks $p \in \mathbb {Z}[X]$ and $p^2-p\equiv 0 {\text {mod}}\langle B(X)\rangle$.

### LPAC and NSS

We have changed our pipeline to generate LPAC proofs instead of PAC proofs, using AMulet 2.0. The experiments are done on the same hardware. In the experiments of this section we only consider Pastèque with the uloop option.

We can only generate NSS proofs to validate the correctness of simple multiplier circuits that don’t require combining algebra and SAT (i.e., extensions). It can be seen in Table 2 that NSS-style LPAC proofs are faster to check for Pacheck 2.0 than NSS proofs for Nuss-Checker. However, the memory usage of Pacheck 2.0 is around an order of magnitude higher than for Nuss-Checker, because Pacheck 2.0 reads and stores the complete constraint set before checking the proof. In Nuss-Checker the constraint set is parsed on the fly.

Pastèque 2.0 is very slow on NSS-style LPAC proofs because it must parse the entire file first, before starting checking, leading to very high memory usage. For those proofs, the uloop has no effect: A full proof step is parsed before checking, but since the entire proof is a single step, it is the same as parsing the full proof beforehand.

LPAC proofs (right block of Table 3) are checked as efficiently as NSS-style LPAC proofs (right block of Table 2) by Pacheck 2.0. For Pastèque 2.0 we gain a significant speed-up when using LPAC proofs. LPAC proofs only need between $1\% - 11 \%$ of the corresponding checking time of NSS-style LPAC proofs. Additionally, checking LPAC proofs is more memory efficient.

If we compare checking LPAC proofs to checking PAC-style LPAC proofs, we can see that both Pacheck 2.0 and Pastèque 2.0 are a factor of two faster on checking LPAC proofs. The memory usage remains the same.

**Table 2:** NSS Proof Checking, without extension (in bold the fastest version)

Multiplier	*n*	Nuss-Checker			LPAC simulates NSS				
		Steps			Steps	Pacheck 2.0		Pastèque 2.0	
			sec	MB		sec	MB	sec	MB
btor	128	1	2	18	1	**2**	98	53	2044
btor	256	1	8	71	1	**7**	385	762	8819
btor	512	1	41	295	1	**35**	1555	14,347	41,712
sp-ar-rc	128	1	3	24	1	**2**	142	80	2845
sp-ar-rc	256	1	13	95	1	**10**	561	1181	12,275
sp-ar-rc	512	1	67	392	1	**48**	2261	21,543	51,415

**Table 3:** LPAC Proof Checking (in bold the fastest version)

Multiplier	*n*	LPAC simulates PAC					LPAC				
		Steps	Pacheck 2.0		Pastèque 2.0		Steps	Pacheck 2.0		Pastèque 2.0	
		($10^6$)	sec	MB	sec	MB	($10^6$)	sec	MB	sec	MB
btor	128	0.3	5	94	14	1305	0.1	**2**	94	7	1305
btor	256	1.3	26	367	67	3467	0.3	**8**	367	37	3816
btor	512	5.2	149	1468	351	14,651	1.0	**37**	1,496	238	16,173
sp-ar-rc	128	0.4	5	137	15	1330	0.1	**2**	137	8	1330
sp-ar-rc	256	1.6	28	543	72	5709	0.6	**11**	543	34	5709
sp-ar-rc	512	6.3	145	2174	381	34,327	2.4	**46**	2173	180	34,327
sp-ar-cl	32	1.6	17	445	88	69 11	0.7	**10**	198	40	2104
sp-dt-lf	32	0.3	2	80	8	857	0.2	**1**	39	4	383
bp-ct-bk	32	0.2	1	54	5	662	0.1	**1**	27	2	268
bp-wt-cl	32	5.5	144	2250	646	36,224	2.4	**88**	1094	292	10,489

We further can see in Table 3 that both Pacheck 2.0 and Pastèque 2.0 are faster on LPAC proofs that simulate PAC than Pacheck 1.0 and Pastèque 1.0 on PAC proofs. The explicit sharing of variables in Pastèque 2.0 also significantly reduces the memory usage, except for sp-ar-rc 512 (the reasons for this behavior are unclear).

Finally, we can compare the performance of Pacheck and Pastèque. In both versions, Pastèque 1.0 and Pastèque 2.0 is less efficient than Pacheck 1.0 and Pacheck 2.0. Pastèque is both much slower and more memory hungry. Verified checkers of SAT certificates [21, 42] have the same level of efficiency as state-of-the-art checkers [53], likely because of the imperative style (unlike our mostly functional code) and the more efficient memory usage by managing most memory directly (e.g., for clauses) instead of relying on the garbage collector.

## Conclusion

In this article we presented the algebraic proof formats PAC, LPAC and NSS, which are able to validate algebraic verification results. We presented soundness and completeness arguments for these proof formats and showed how proof certificates can be generated as a by-product of algebraic reasoning on the use case of arithmetic circuit verification. Proofs in NSS capture whether a polynomial can be represented as a linear combination of a given set of polynomials by providing the co-factors of the linear combination. PAC proofs dynamically capture whether a polynomial can be derived providing a sequence of proof steps. We extend PAC by including an extension rule capturing rewriting techniques. Furthermore, we added a deletion rule and used indices for polynomials. Our novel format LPAC extends PAC by providing the ability to combine several steps at once.

Our proof checkers Pacheck, Pastèque, and Nuss-Checker are able to check proofs efficiently. Our experiments showed that the PAC optimizations cut the memory usage of Pacheck in half and reduce the runtime by around 30–80%. Our reimplementation Pacheck 2.0 and Pastèque 2.0, which use LPAC further reduce the runtime by around 25–50%. To our surprise, the size of NSS proofs does not explode in our experiments and is faster to check than PAC. This was the motivation to combine the advantages of PAC and NSS into LPAC. Checking LPAC proofs is as time efficient as checking NSS proofs, while still providing detailed error messages. However, the memory usage of checking LPAC proofs is an order of magnitude higher than checking pure NSS proofs. On LPAC, Pacheck was three times faster than Pastèque and used an order of magnitude less memory, whereas Pastèque was formally verified in Isabelle.

In the future we want to capture more general extension rules in PAC as the calculus from Sect. 2 allows. We imagine that it can be extended in two ways. First, we could relax the condition $p^2 = p$. This condition is necessary to have $v^2=v$, but could be lifted even if it means that $v^n$ cannot be simplified to *v* anymore, requiring to manipulate exponents. Second, we currently restrict the extension to the form $v=p$ where *p* contains no new variables. The correctness theorem does not rely on that and we leave it as future work to determine whether lifting one of these restrictions can lead to shorter proofs.

In AMulet 2.0 no redundant proof steps are generated, hence no backward proof checking is necessary unlike SAT certificates. This might still be interesting in other applications. Another idea for future work is to bridge the gap between C and Isabelle, either by imperative code or by verifying the C code directly.

## Supplementary Information

Below is the link to the electronic supplementary material.

Supplementary file 1 (pdf 20 KB)

Supplementary file 2 (pdf 20 KB)

Supplementary file 3 (pdf 6 KB)

Supplementary file 4 (pdf 23 KB)

Supplementary file 5 (pdf 23 KB)

## Footnotes

1 The hash map setup relies on exceptions, which is why we did not do that for Pastèque 1.0, but now we changed the setup.
