# Supplementary figures and images for: Practical algebraic calculus and Nullstellensatz with the checkers Pacheck and Pastèque and Nuss-Checker
Source: Form Methods Syst Des. 2022 Apr 11;64(1-3):73–107. doi: 10.1007/s10703-022-00391-x (PMC11682020; doi:10.1007/s10703-022-00391-x)

Memory usage in MB

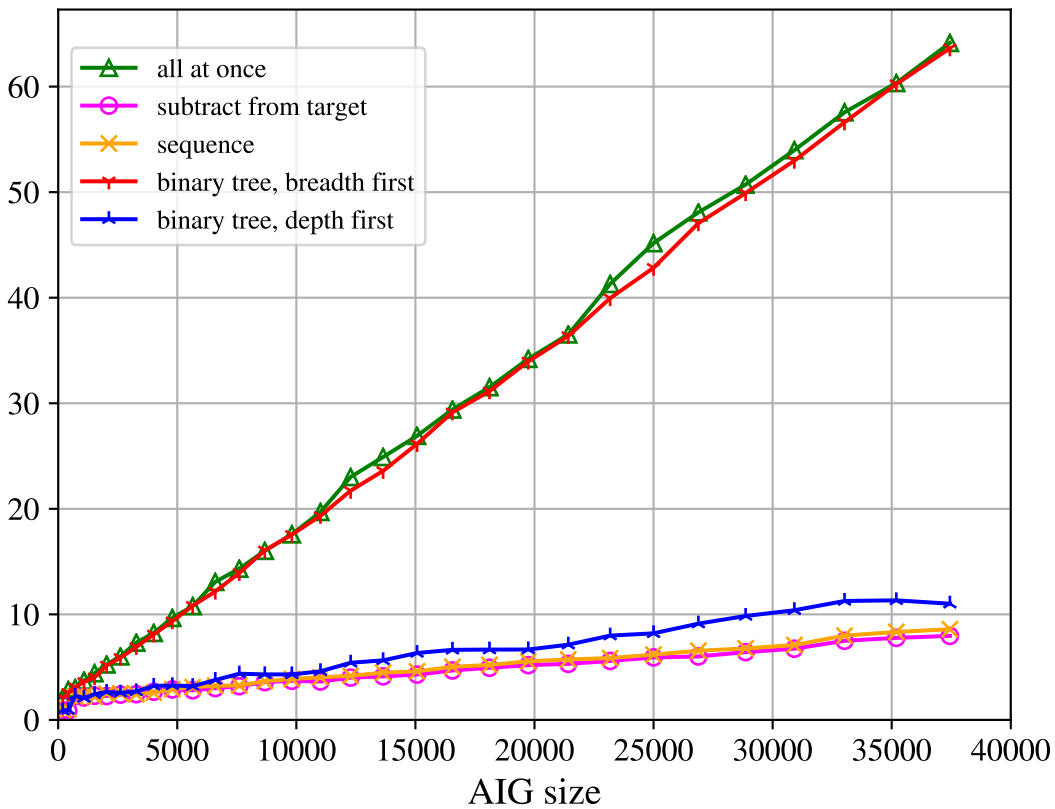

Supplement: Supplementary file 1 — (pdf 20 KB) [file 10703_2022_391_MOESM1_ESM.pdf]

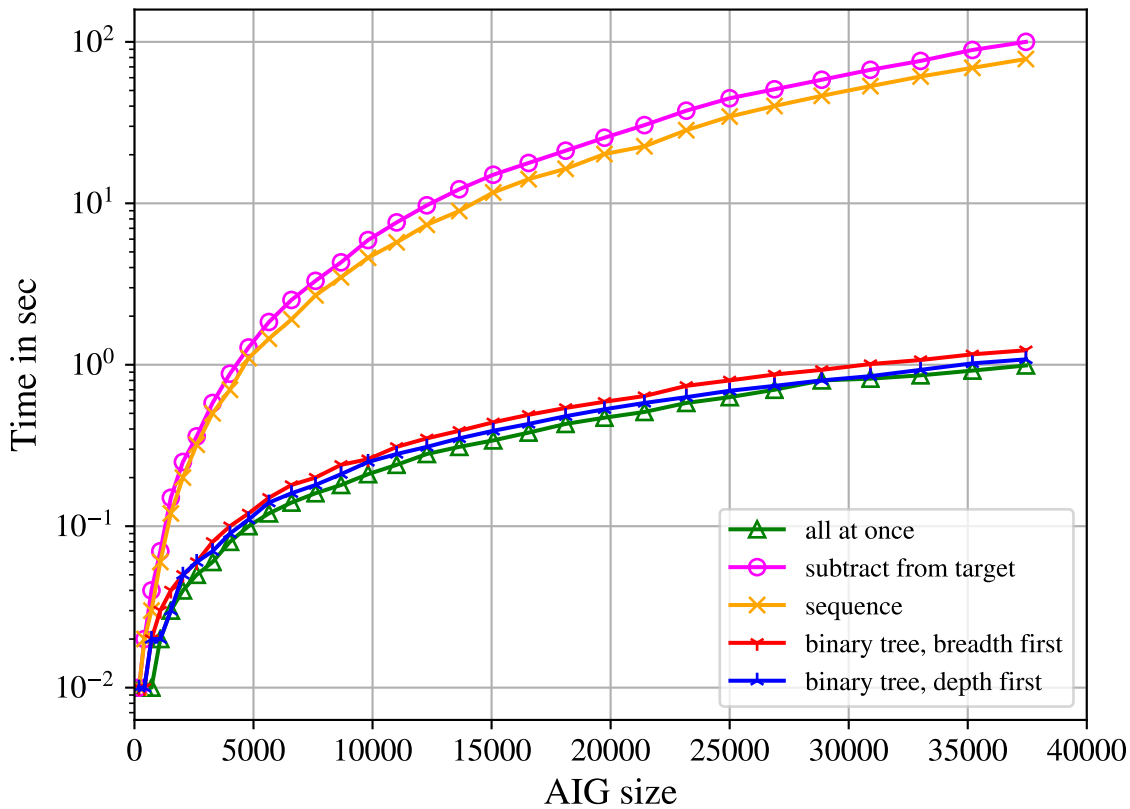

Supplement: Supplementary file 2 — (pdf 20 KB) [file 10703_2022_391_MOESM2_ESM.pdf]

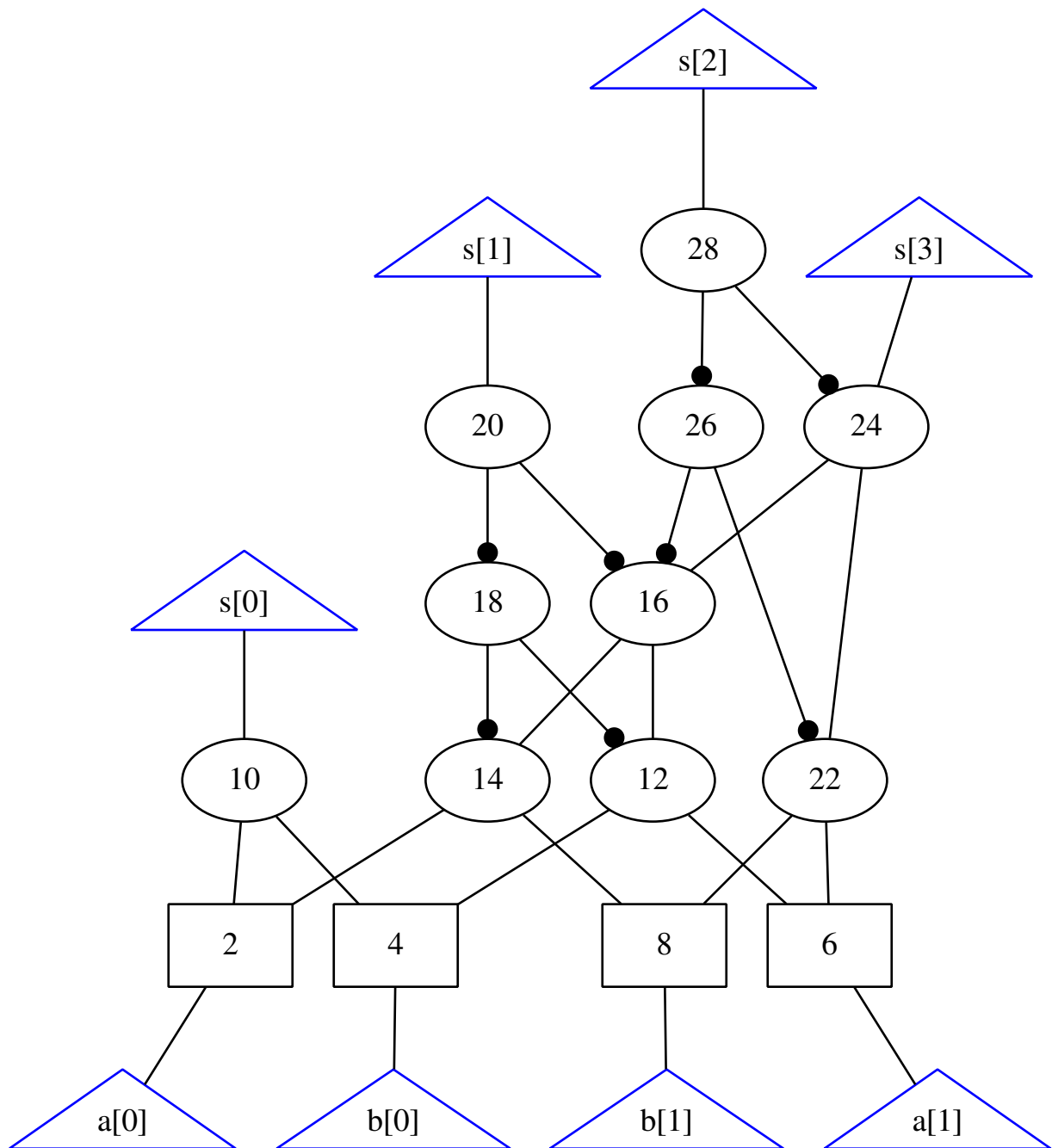

Supplement: Supplementary file 3 — (pdf 6 KB) [file 10703_2022_391_MOESM3_ESM.pdf]

Memory usage in MB

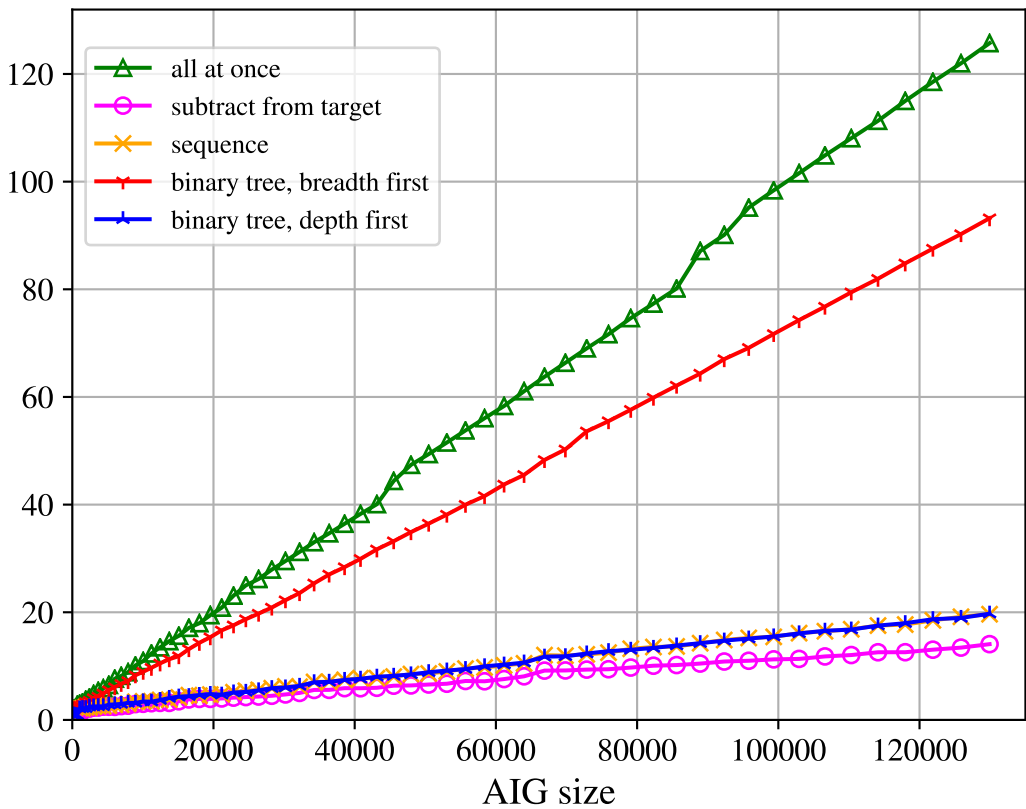

Supplement: Supplementary file 4 — (pdf 23 KB) [file 10703_2022_391_MOESM4_ESM.pdf]

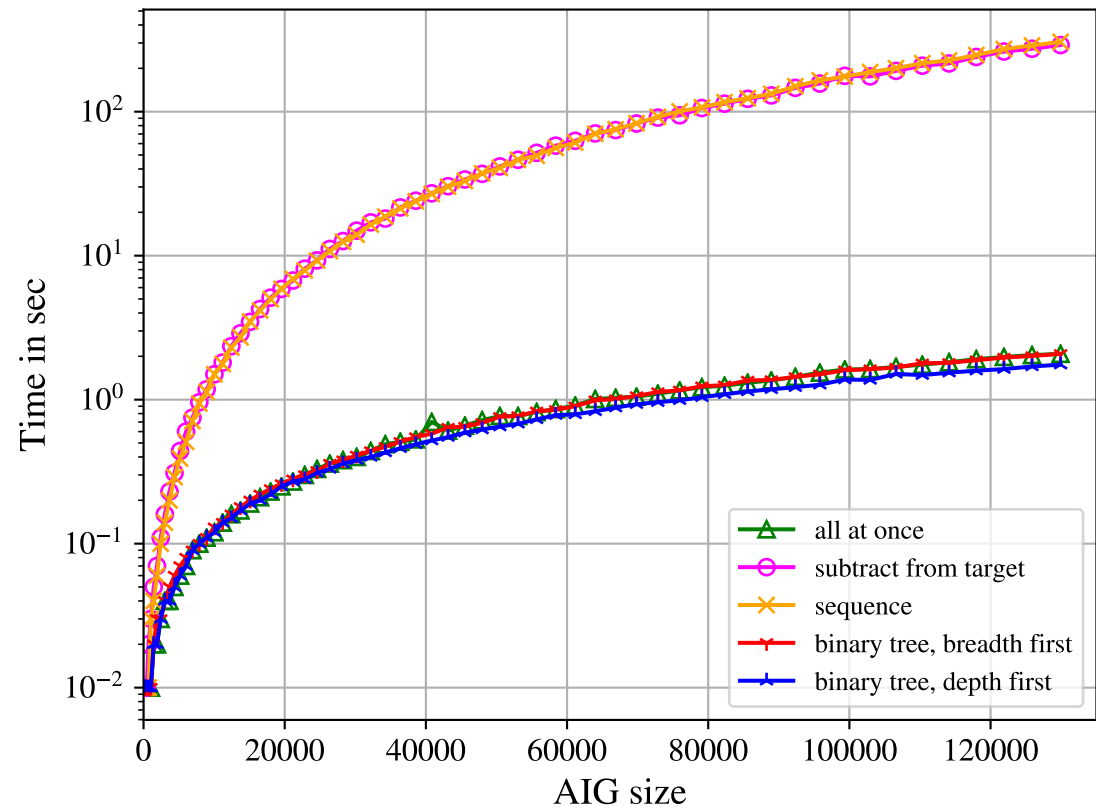

Supplement: Supplementary file 5 — (pdf 23 KB) [file 10703_2022_391_MOESM5_ESM.pdf]
